# A prospective framework for testing whether pairwise transcriptomic module coordination can represent drought-associated root architectural change

**DOI:** 10.3389/fpls.2026.1965677

**Published:** 2026-10-16

**Authors:** Xiaochen Liu

**Affiliations:** Department of Science, The University of Melbourne, Parkville, Melbourne, VIC, Australia

**Keywords:** co-expression networks, drought stress, falsifiability, generalized contrastive principal component analysis, interaction order, root plasticity, root system architecture (RSA), transcriptomics

## Abstract

Soil drying changes the root tissue distribution and amount in the soil, while the shoot limits water loss. The root length and surface area describe how extensively roots explore and contact the soil. The root volume and average diameter reflect tissue investment and radial growth. Together, these traits show only the final architectural outcome. They do not tell us whether the change comes from plastic adjustment, slower development, or damage. Bulk root transcriptomes further complicate interpretation by combining signals from tissues, developmental zones, and cell types. We still do not know whether drought-related changes in the root architecture can be represented by a relatively simple coordination among the transcriptomic modules or whether more complex combinations are needed. We therefore propose a prospective framework to test whether module eigengenes and their pairwise products adequately predict root architectural change. Root architectural deformation (RAD) is defined as a candidate geometric coordinate from four fixed traits using generalized contrastive principal component analysis (gcPCA) after pooled common standardization. Its variance ratio objective avoids manual choice of the contrast parameter used in classical contrastive principal component analysis (cPCA). Testing still depends on Clause Zero: phenotype and transcriptome measurements must be linked to an independently measured soil water status and collected within a physiologically appropriate window. Module eigengenes and their pairwise products form the specified predictor basis. Topology-based weighting, locked least absolute shrinkage and selection operator (LASSO), and matched target-decoy screening narrow the candidate set. Clause A asks whether the retained terms rise above a structured-permutation ceiling. Clause B asks whether a pre-specified higher-capacity model recovers out-of-sample predictive information beyond this basis, independently of candidate survival. Public wheat phenotypes show cPCA sensitivity and favorable local gcPCA diagnostics, but do not establish a stable plasticity phenotype. Controlled cases illustrate intended readouts. However, the full empirical framework remains untested. A meaningful gain from the higher-capacity model would argue against pairwise practical adequacy. Failure to determine a clear advantage would not, by itself, establish pairwise adequacy. A non-inferiority analysis against a prospectively justified utility margin would still be necessary. Biological interpretation comes later and would depend on independent validation, root tissue localization, perturbation experiments, and evidence across genotypes, environments, and yield.

## 1 Introduction

Declining soil water potential constrains both root hydraulics and growth. During gradual drying, roots may adjust the growth, branching, and radial tissue allocation before turgor loss or injury occurs. These adjustments change the soil volume explored and the surface available for absorption. Root architecture can therefore connect local growth responses with whole-plant water management. However, acclimatization, delayed development, and injury can produce similar final structures. Transcriptomic studies need measured water availability and a defined physiological sampling window to distinguish these interpretations (Koevoets et al., 2016; Lynch, 2022; Ranjan et al., 2022).

The rice gene DEEPER ROOTING 1 (*DRO1*) provides a direct example of the link between architecture and drought tolerance. Introducing *DRO1* changed the root growth angle and increased rooting in deeper soil. Under the drought conditions tested, the resulting line showed better drought avoidance and higher yield than the shallow-rooting parent (Uga et al., 2013). The benefit of an architectural trait thus depends on the soil depth, developmental stage, and drought regime.

At the whole-root scale, the length and surface area describe the exploration and absorptive extent, while the volume and mean diameter describe the tissue investment and radial construction. These four traits provide a deliberately sparse description of architectural change. They do not resolve the anatomy, developmental history, hydraulic conductivity, branching, or cellular composition. Their role is to define a macroscopic prediction target before fitting transcriptomic models.

Bulk root expression has a similar interpretive difficulty. A sample combines the root cap; the meristematic, elongating, and mature cells; and the cortex and vascular tissues. Drought can change both their expression and their relative abundance. Co-expression modules and eigengenes summarize part of this variation (Kitano, 2002; Barabási and Oltvai, 2004). Projecting onto a fixed reference gives treatments common eigengene coordinates, but with their biological comparability still needing independent evidence. Studies of wheat landraces have identified drought-responsive genes, modules, and module–trait associations (Barratt et al., 2023). These associations leave open how complex a predictor must be to represent root architectural change.

Systems genetics relates coordinated molecular states to phenotypic variation (Civelek and Lusis, 2014). Deep learning and graph-based models can capture nonlinear relationships and combine multiple omics layers (Wang et al., 2026b). This flexibility raises a useful question for root biology. Given comparable module states and a fixed architectural target, how much predictive complexity is needed? We ask whether eigengenes and their pairwise Hadamard products perform within a pre-specified practical margin of a higher-capacity model. The comparison concerns this predictor basis and fitting procedure rather than the general interaction order.

The framework proceeds from root architectural deformation (RAD) geometry and Clause Zero to module continuity, a pairwise predictor basis, Clauses A and B, and biological follow-up (Platt, 1964; Nosek et al., 2018). The evidence used here has three distinct roles. **Figures 1**–**3** and **Supplementary Figures S1**–**S3** describe the public phenotypes, including the species screen, source precision analysis, and durum reference. **Figures 4**–**10** combine controlled computations, such as topological overlap matrix (TOM) estimation and least absolute shrinkage and selection operator (LASSO) resampling, with constructed displays, including the scores in **Figure 8** and the errors and attributions in **Figure 9**. **Supplementary Figure S4** and **Supplementary Table S8** report a separate conditional simulation study of Clause B intervals and an independent test set size for frozen predictors under specified error models (**Supplementary Methods S7.5**). The study evaluates the conditional procedure, not the end-to-end Clause A, target-decoy screening, or the whole workflow. No Clause Zero-compliant matched phenotype–transcriptome dataset is analyzed. The full empirical Clause A procedure, empirical Clause B nested training and evaluation, experiment-specific utility margin, and raw trait measurement error propagation remain prospective.

**Figure 1:**
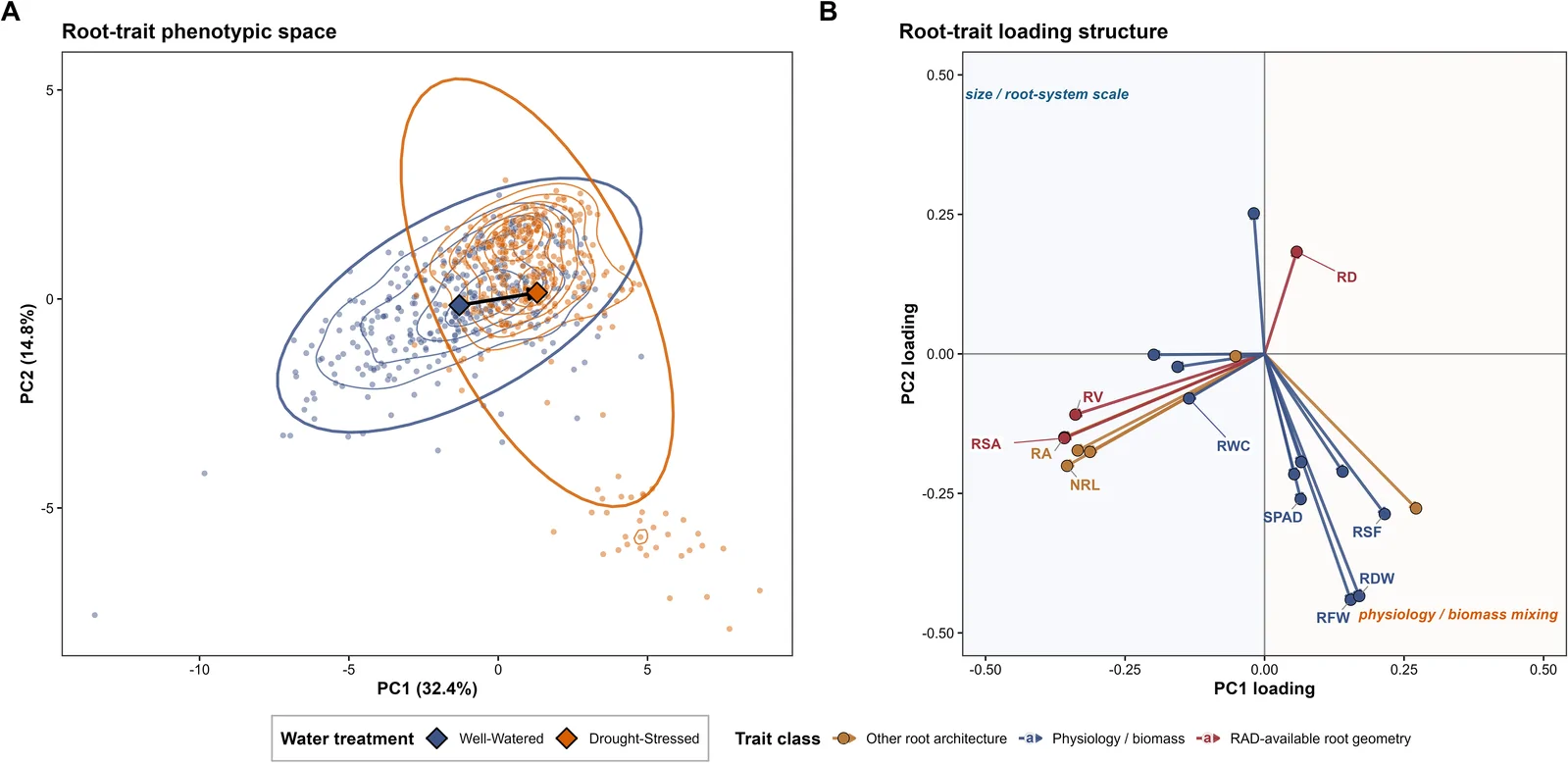
Drought phenotype variation in root system architecture. **(A)** An unsupervised principal component analysis (PCA) has been carried out using 20 physiological and root architectural parameters of the whole dataset (*N* = 720; drought: *n* = 360; well-watered: *n* = 360). *Points* are measurements, plotted in color code according to treatment. The contours and centroids of both cohorts are represented in the PC1–PC2 plane. PC1 and PC2 explain 32.4% and 14.8% of the variance, respectively. **(B)** Traits correlated with PC1 and PC2 have been sorted based on root architectural deformation (RAD) geometry, non-RAD root system, and physiological/biomass traits. The separate source field RA (−0.359), RSA (−0.359), and NRL (−0.354) were the traits most correlated with PC1. The traits with the highest absolute values correlated with PC2 were root fresh weight (RFW = −0.441), root dry weight (RDW = −0.434), and the fresh weight root/shoot ratio (RSF = −0.287). RSA here is a source field label, not an abbreviation for root system architecture. **Supplementary Table S4** records its unresolved physical mapping. The axes created by PCA from 20 traits describe the root system size and physiological/biomass state. There is no stress axis observed physically, and the four-trait RAD target is left at its original position. Based on our analyses of the dataset published by Pour-Aboughadareh and Poczai, 2021b.

**Figure 2:**
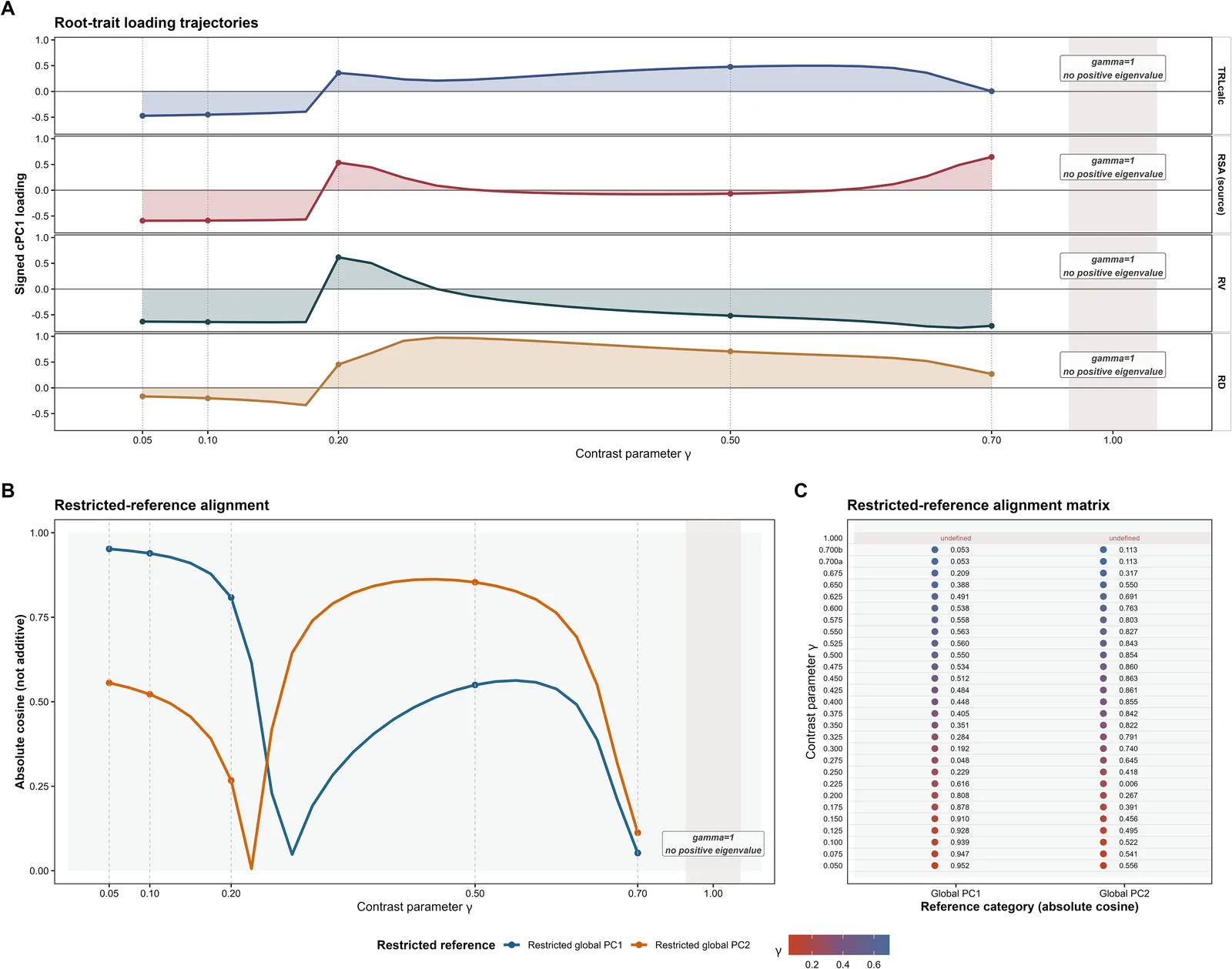
Contrast modification by manual choice affects the candidate root architecture coordinate system. Both treatments in the archived 720-record branch use one pooled standardization before covariance estimation and score projection. **(A)** Signed loading paths for TRLcalc, RSA (source field), root volume (RV), and root diameter (RD). TRLcalc is the operational SRL × RDW product after the archived additional rounding, with unresolved physical units. RD replaces the legacy ARD display alias without implying the mean diameter. All archived *γ* values and sign conventions are retained, with *γ* increasing from 0.05 to 1.00 on the horizontal axes. At *γ* = 1, no positive additive-contrast eigenvalue exists and alignment is undefined. At *γ* = 0.70, loadings are RSA = 0.646, RV = −0.715, RD = 0.269, and TRLcalc = 0.003. **(B)** Separate absolute cosines with global PC1 and PC2 after restricting both coefficient vectors to the shared RSA/RV/RD fields and normalizing their Euclidean lengths in the common pooled-SD metric. The non-equivalent NRL/TRLcalc coordinates are excluded. PC1/PC2 cosines are 0.053/0.113 at *γ* = 0.70, 0.550/0.854 at *γ* = 0.50, and 0.952/0.556 at *γ* = 0.05. **(C)** The same values appear in a *γ*-by-reference alignment matrix. *Columns* are reference categories, not orthogonal coordinates. *Cells* print absolute cosines and *point color* identifies *γ*. The two stored near-0.70 entries are retained as 0.700a/b, and the *γ* = 1 row is marked undefined. The references remain non-orthogonal; thus, their cosines are non-additive and do not represent variance shares. Based on our analyses of the dataset published by Pour-Aboughadareh and Poczai, 2021b.

**Figure 3:**
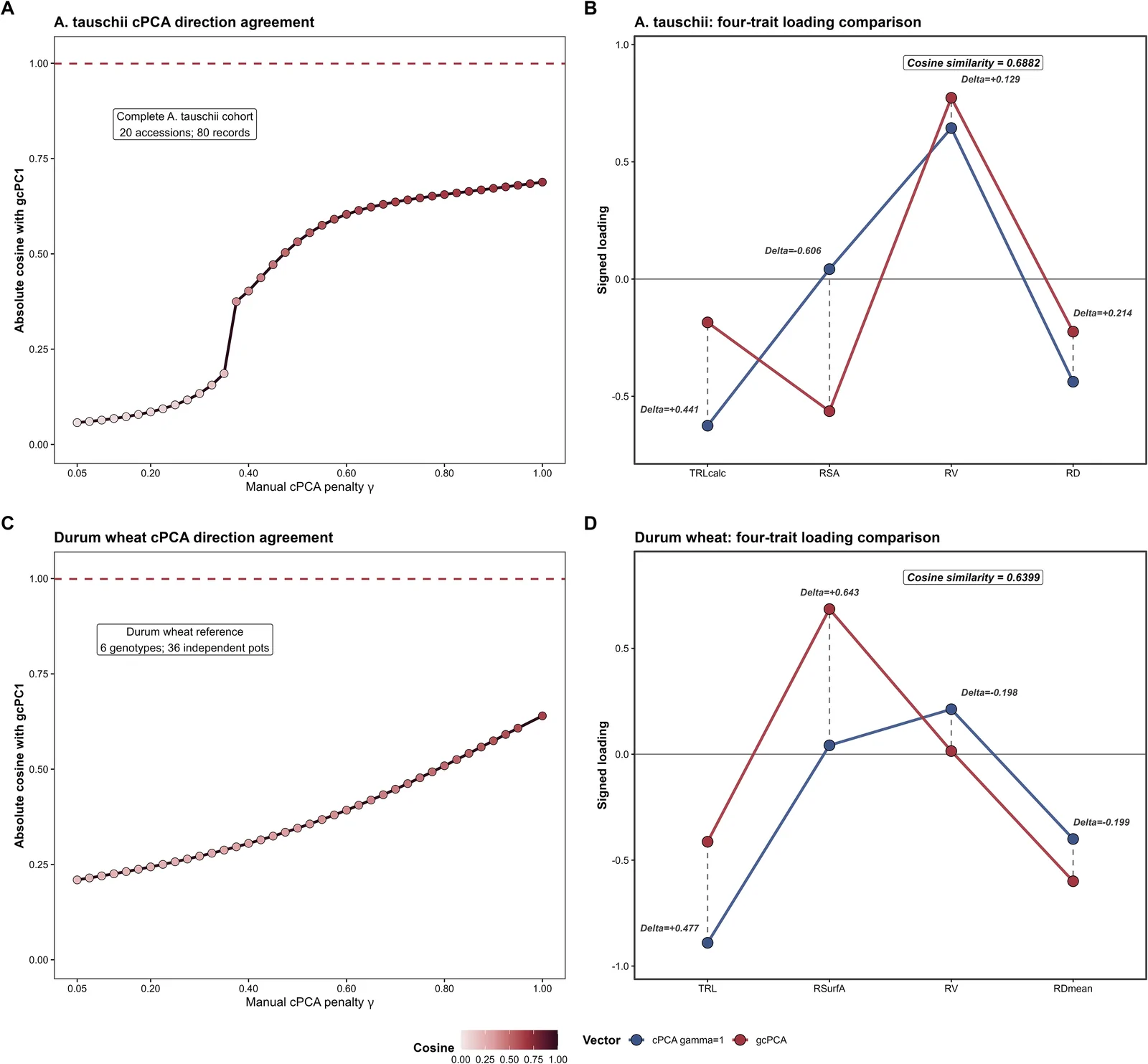
Matched contrastive principal component analysis (cPCA) and generalized cPCA (gcPCA) geometric comparisons in two single-species cohorts. **(A, C)** Absolute cosine between the first cPCA direction at each sampled *γ* and the first gcPCA direction. *Lines* retain the frozen gamma registries. The *dashed 0.999 line* is a display reference. **(B, D)** Native pooled-standardized unit loadings for cPCA at *γ* = 1 (*blue*) and gcPCA (*wine*). Delta = gcPCA − cPCA. The cPCA reference is positive on the source RSA field (RSurfA in durum), and gcPCA is sign-aligned to it. **(A, B)** All 80 *Aegilops tauschii* records from 20 accessions, with 40 per treatment and two recorded replications, use source precision. TRLcalc = SRL × RDW is operational, with unresolved physical units. RSA and RD retain their source identities (Based on our analyses of the dataset published by Pour-Aboughadareh and Poczai, 2021b). **(C, D)** Durum wheat: six genotypes and 36 independent pots, three per genotype and treatment (Based on the independent durum wheat dataset of Urbanavičiūtė et al. 2022b). *TRL*, *RSurfA*, *RV*, and *RDmean* are the directly measured length (in centimeters), surface area (in square centimeters), volume (in cubic centimeters), and mean diameter (in millimeters). These measurements are not interchangeable with the *A. tauschii* operational fields. The *γ* = 1 absolute cosines are 0.688172 and 0.639882, respectively. The fixed gcPCA ridge is 10^−6^. This frozen range comparison is not an optimized cPCA performance benchmark. Display signs do not alter the frozen uncertainty analyses (**Supplementary Figure S3**; **Supplementary Tables S6**, **S7**).

**Figure 4:**
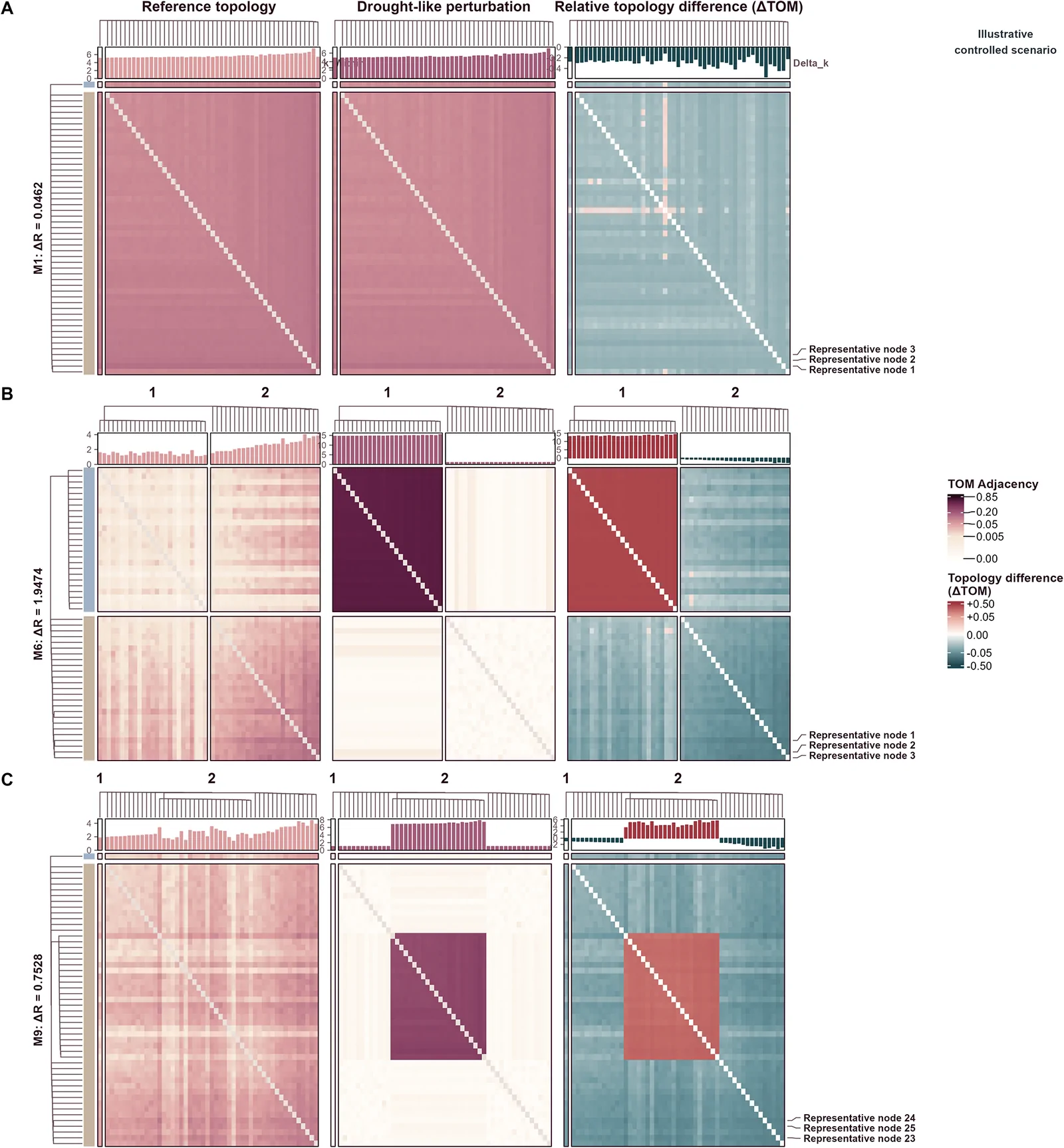
Relative topology deviations in constructed co-expression modules. The controlled topology case contains n=150 samples per condition and p=770 features in 10 predefined modules of sizes 150, 120, 100, 90, 80, 50, 40, 30, 60, and 50. Networks use unsigned adjacency with power β=6. The display exponent 0.20 transforms the heatmap values after topological overlap matrix (TOM) estimation. It is not a network construction parameter. **(A)** Reference, foreground, and topology difference matrices for M1 ( ΔR=0.0462). **(B)** M6 ( ΔR=1.9474). **(C)** M9 ( ΔR=0.7528). Each heatmap shows 50 genes, selected by maximum connectivity across conditions when the module is larger, with a common average linkage order within that module. The $k_{\text{Within}}$ bars are displayed sub-matrix column sums, including unit diagonals, with Δk as their difference. ΔR uses the complete module and untransformed TOMs, including the diagonal in the denominator (**Supplementary Methods S2.3**). The figure-level label applies to all panels: TOMs were estimated in this controlled scenario, and no empirical transcriptomic model was fitted.

**Figure 5:**
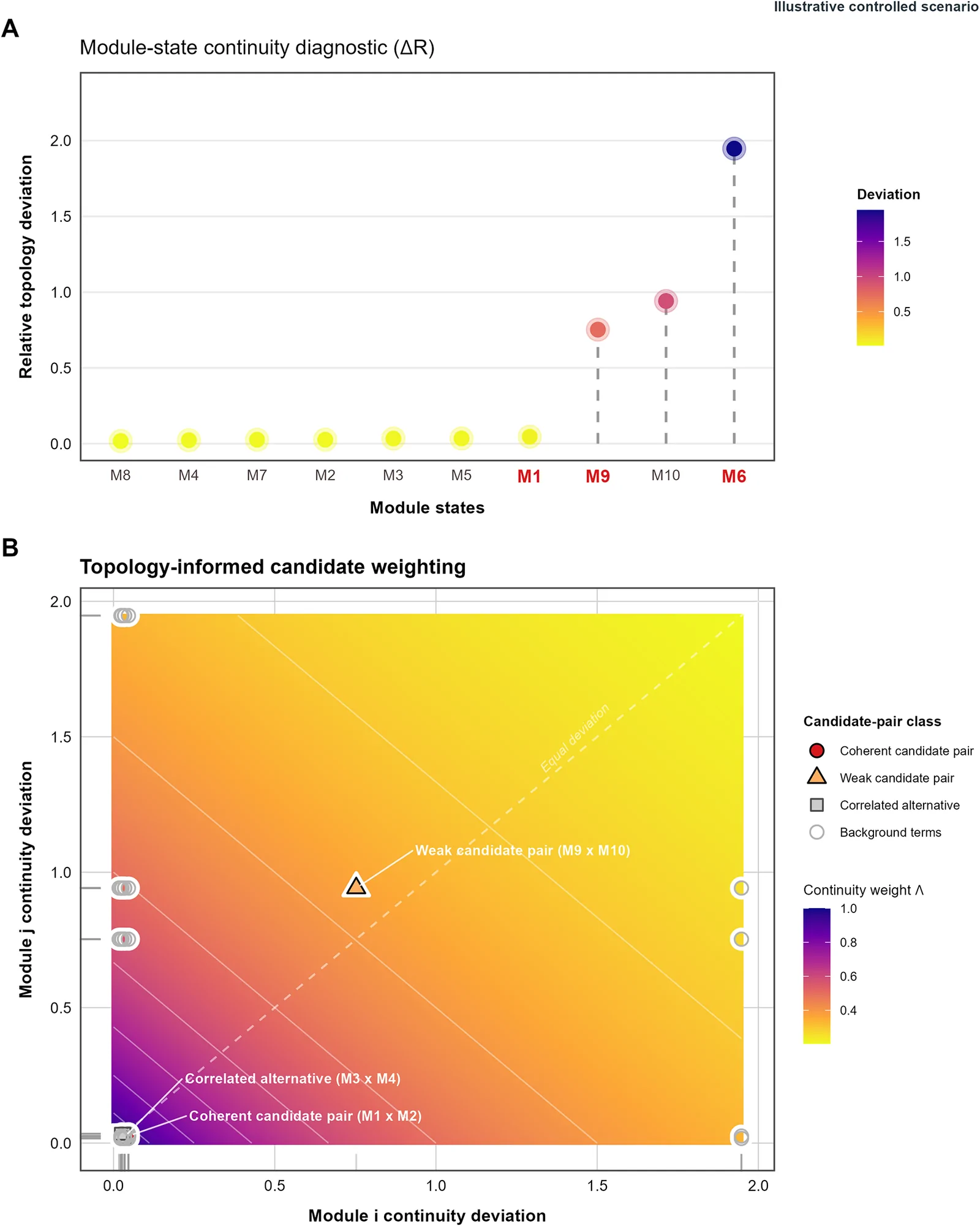
Weights applied to constructed module pair comparisons. **(A)** Full-matrix relative topology deviation Δ*R* for the 10 frozen gene modules, ranging from 0.0174 to 1.9474. M1, M6, and M9 match **Figure 4**. Categorical reorganization labels have been removed. **(B)** The *background color* and *contours* show the continuity weight Λ = 1/(1 + $\Delta R_{i}$ + $\Delta R_{j}$). *Point positions* are the corresponding module deviations. The inverse weight 1/Λ is the assigned penalty multiplier: 1.0723 for M1 × M2, 2.6943 for M9 × M10, and 1.0567 for the correlated alternative M3 × M4. Its low penalty shows why topology weighting alone does not remove collinearity. The weights follow the archived module index registry. The separate eigengene column convention used in screening remains described in **Supplementary Methods S2.3**. The figure-level label applies to both controlled panels, and no empirical transcriptomic model was fitted.

**Figure 6:**
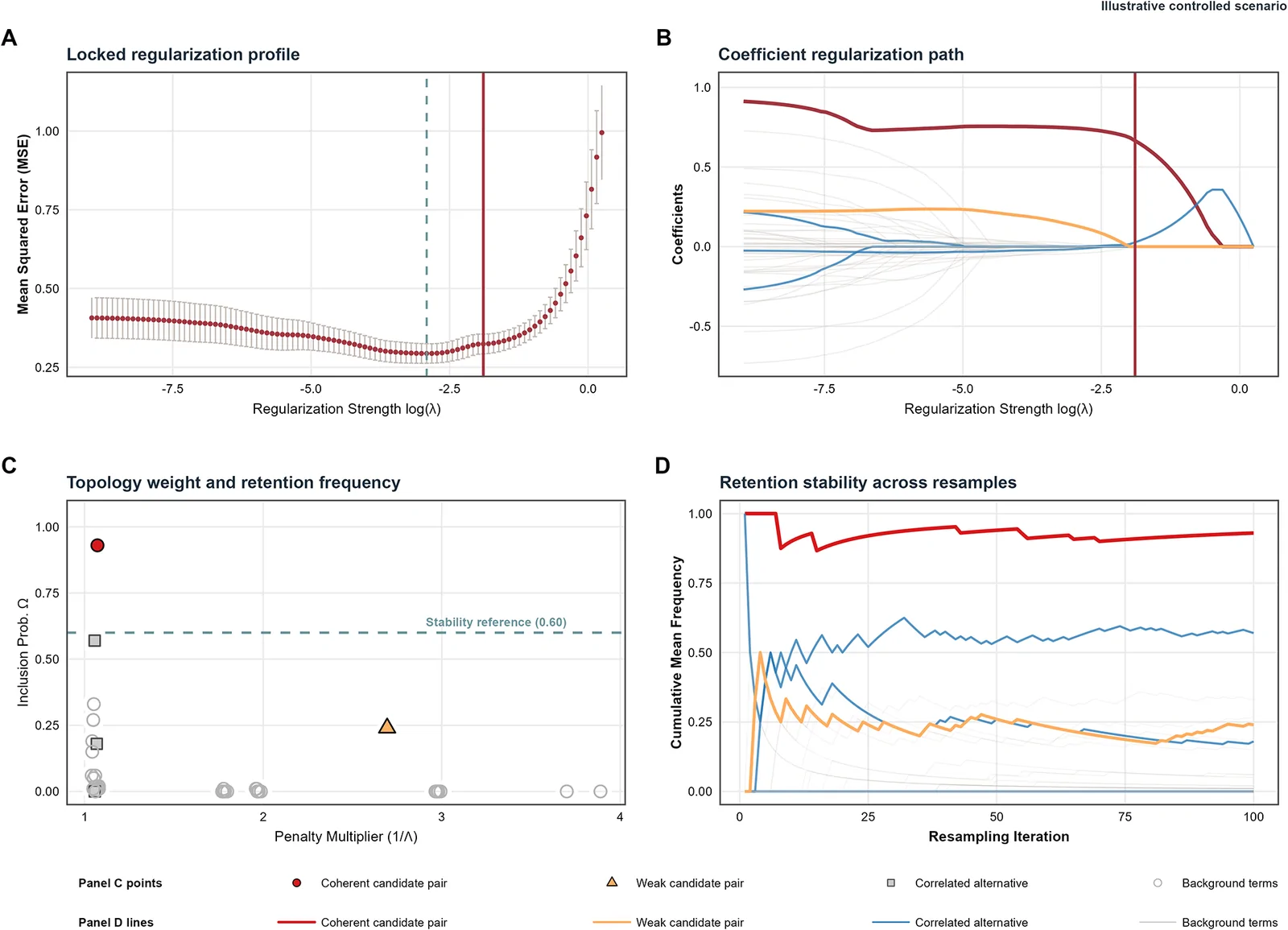
Locked-LASSO (least absolute shrinkage and selection operator) fitting and resampling in a configured candidate screen. **(A)** The 10-fold cross-validation profile with $\lambda_{\text{min}}$ = 0.0542 and the locked $\lambda_{1\text{se}}$ = 0.1509. **(B)** Coefficient paths across the regularization grid. **(A, B)** use the original predictor matrix, while **(C, D)** show the original feature retention in the augmented target-plus-matched decoy fits at the same locked $\lambda_{1\text{se}}$. **(C)** Retention frequency Ω against the topology-derived penalty multiplier, with the operational reference Ω ≥ 0.60. **(D)** Cumulative retention over 100 subsamples, each containing 50% of observations without replacement. The final frequencies are 0.93 for M1 × M2, 0.24 for M9 × M10, and 0.57 for M3 × M4, matching **(C)** The figure-level label applies to all panels: these are configured LASSO fits and resampling results, subject to the eigengene label limitation in **Supplementary Methods S2.3**. No empirical transcriptomic model was fitted.

**Figure 7:**
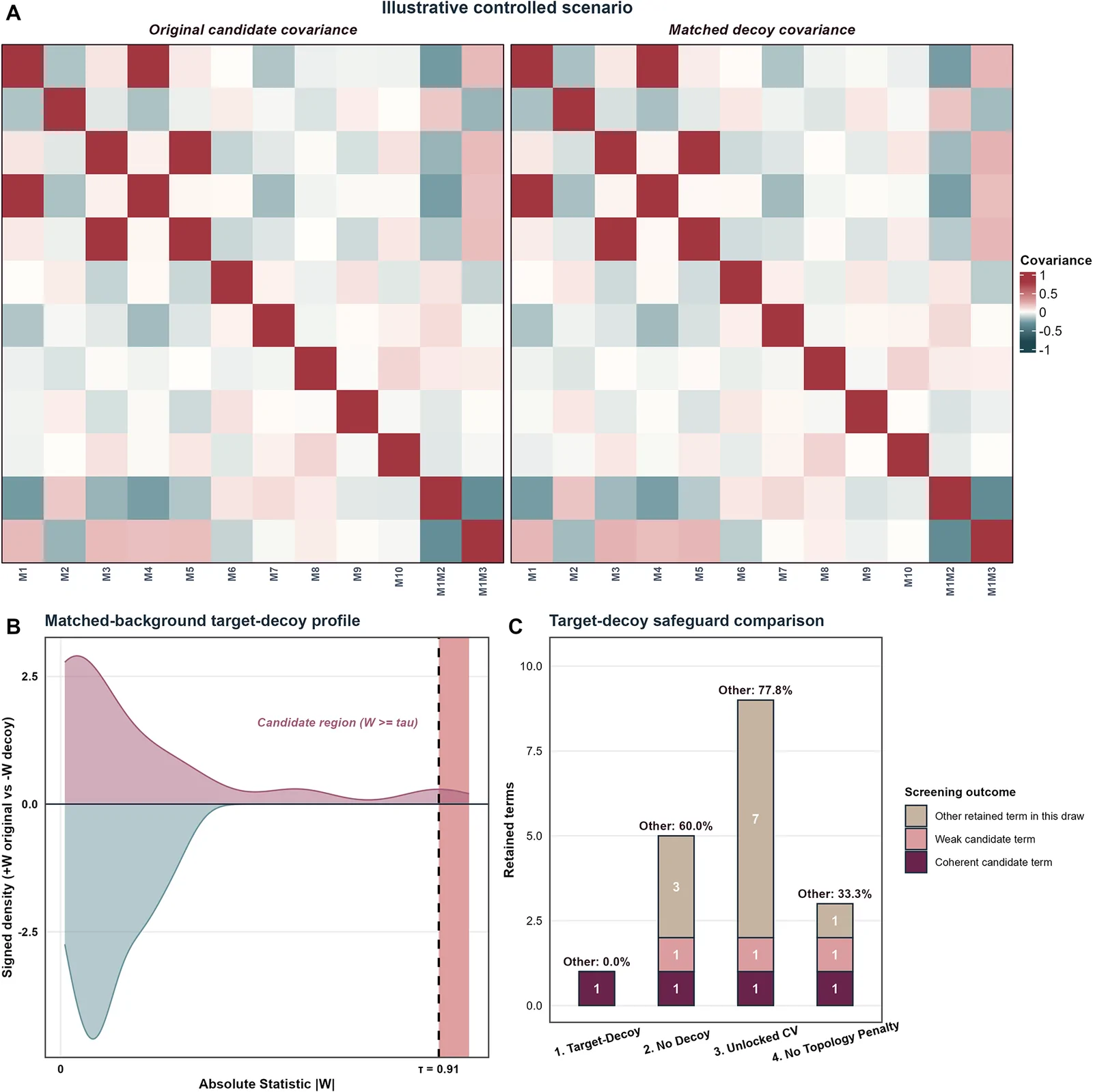
Matched target-decoy screening in the controlled candidate subset. **(A)** Covariance structures between the original and matched-decoy feature vectors in the controlled target-decoy setting. Similarity is suggestive, but does not imply exchangeability of Model-X. **(B)** Density of the original and decoy screening statistics at the display threshold value *τ* = 0.910. The *shaded* candidate area represents the display protocol for this particular realization. **(C)** Candidate retention at the 25% baseline inclusion benchmark. The No Penalty, Unlocked CV, and No Decoy methods retain a higher proportion of the correlated candidates, while the topology-informed target-decoy screen retains the pre-specified coherent term in this particular realization. The figure-level label applies to all panels. No empirical transcriptomic model was fitted.

**Figure 8:**
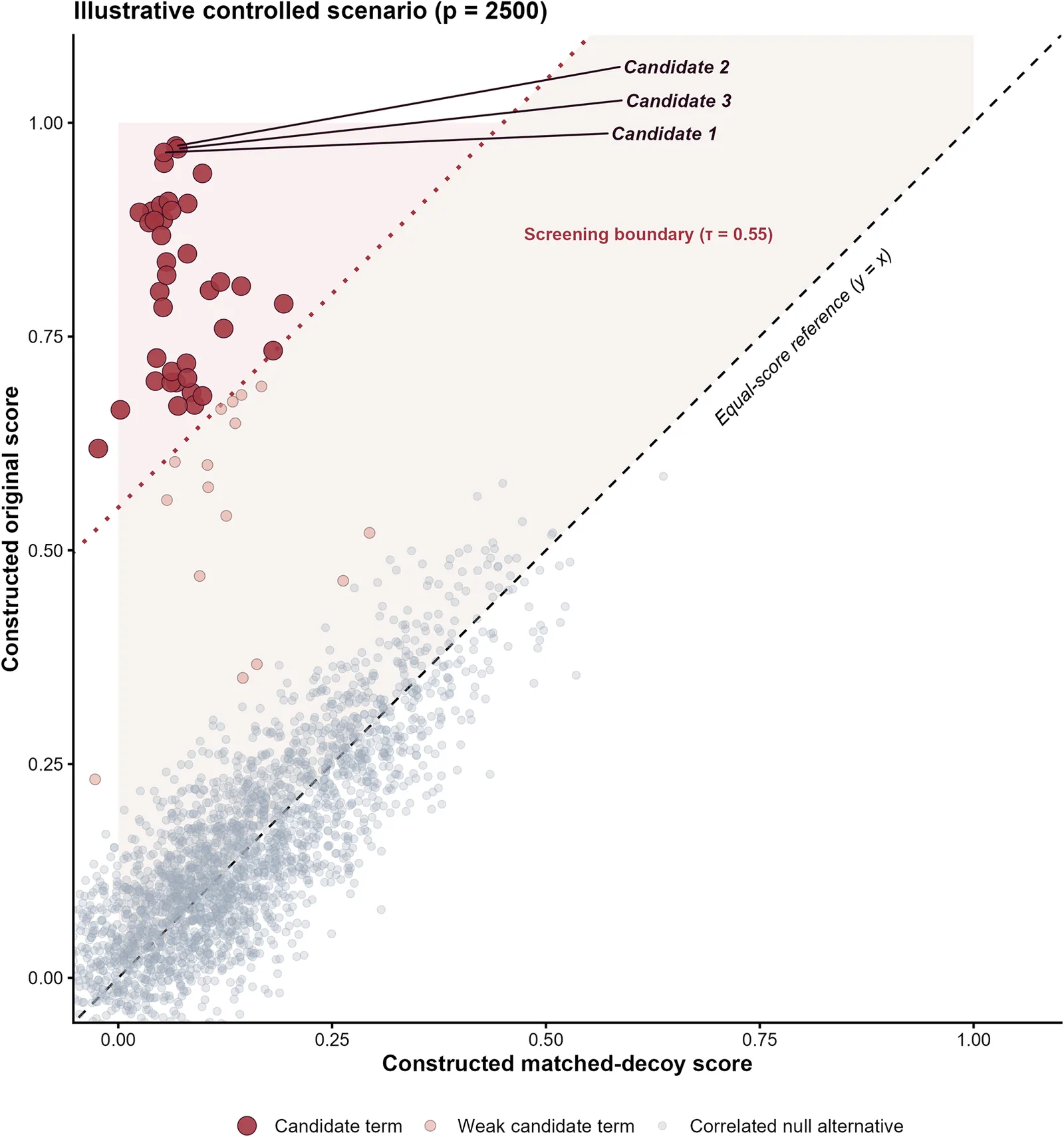
Triaging of the target–decoy pairs for constructed terms. The figure-level label identifies the directly generated original and decoy scores on explicitly labeled constructed score axes; they are not estimated inclusion frequencies. The nominal threshold is *τ* = 0.550 and forms the region where the candidates are located. The *dashed line* indicates equality between the original and decoy scores. At *τ* = 0.550, 39 terms fall in the nominal candidate region, 16 in the weak status region, and 2,445 in the null status region. These screening regions are reported separately from the underlying signal classes. No empirical transcriptomic model was fitted.

**Figure 9:**
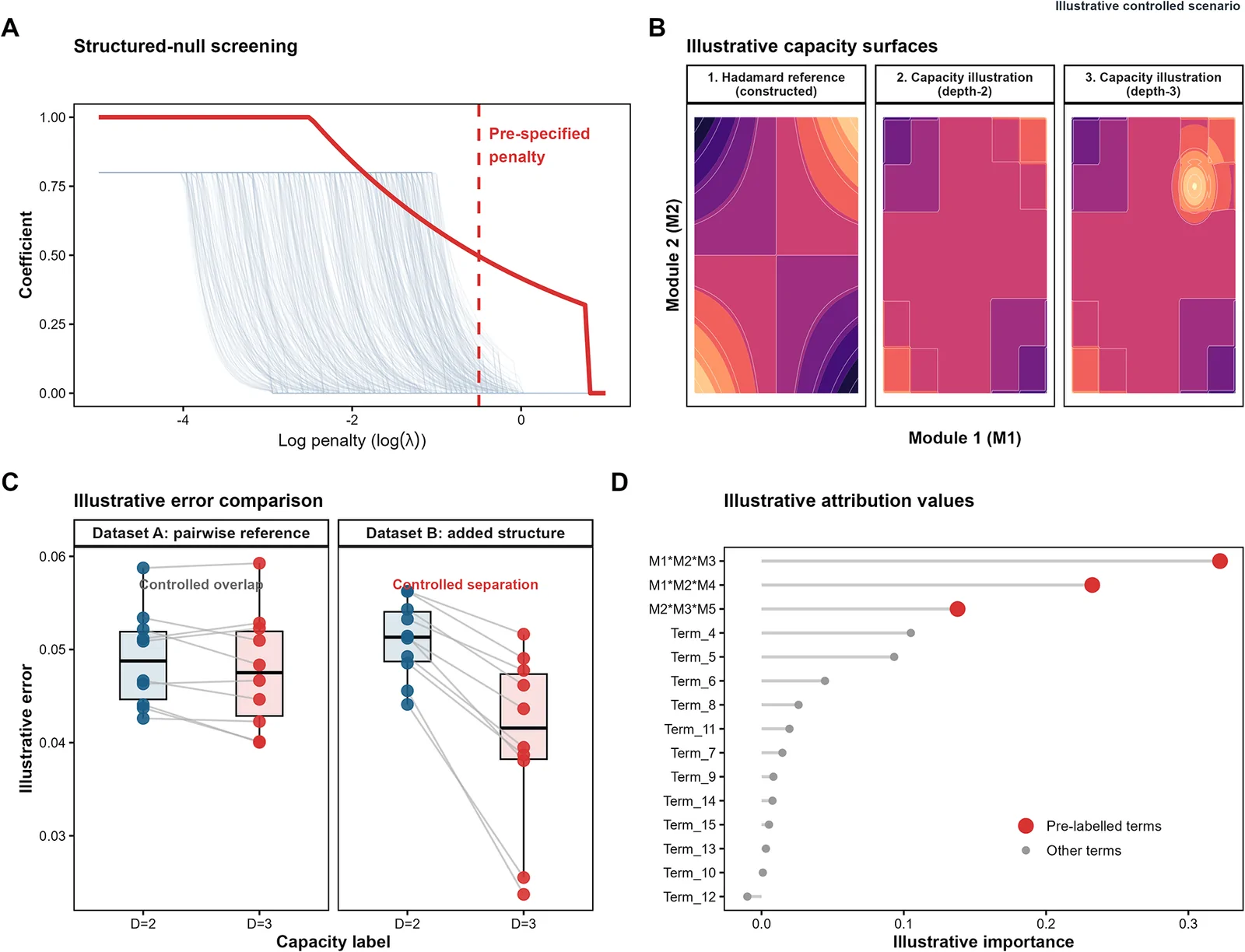
Constructed candidate persistence and model capacity readouts. **(A)** Constructed coefficient trajectories illustrate a candidate persistence question under a fixed penalty; they are not fitted permutation paths. **(B)** Directly specified surfaces show a Hadamard reference and schematic capacity contrasts labeled depth 2 and depth 3. Neither surface is a fitted tree, and depth 2 is not the Equation 6 predictor. **(C)** The 10 constructed error pairs per scenario are joined by a display identifier. *Boxes* show medians and interquartile ranges, with *whiskers* extending to the most extreme values within 1.5 interquartile ranges. The values are directly generated, not held-out errors, independent folds, or model uncertainty estimates. The illustrative error scale does not define δ*. **(D)** Directly assigned importance values emphasize pre-labeled terms, and no model attribution or Shapley additive explanations (SHAP) analysis was performed. The figure-level label applies to all four constructed panels. No empirical transcriptomic model was fitted. These values provide no empirical Clause B or interaction order evidence.

**Figure 10:**
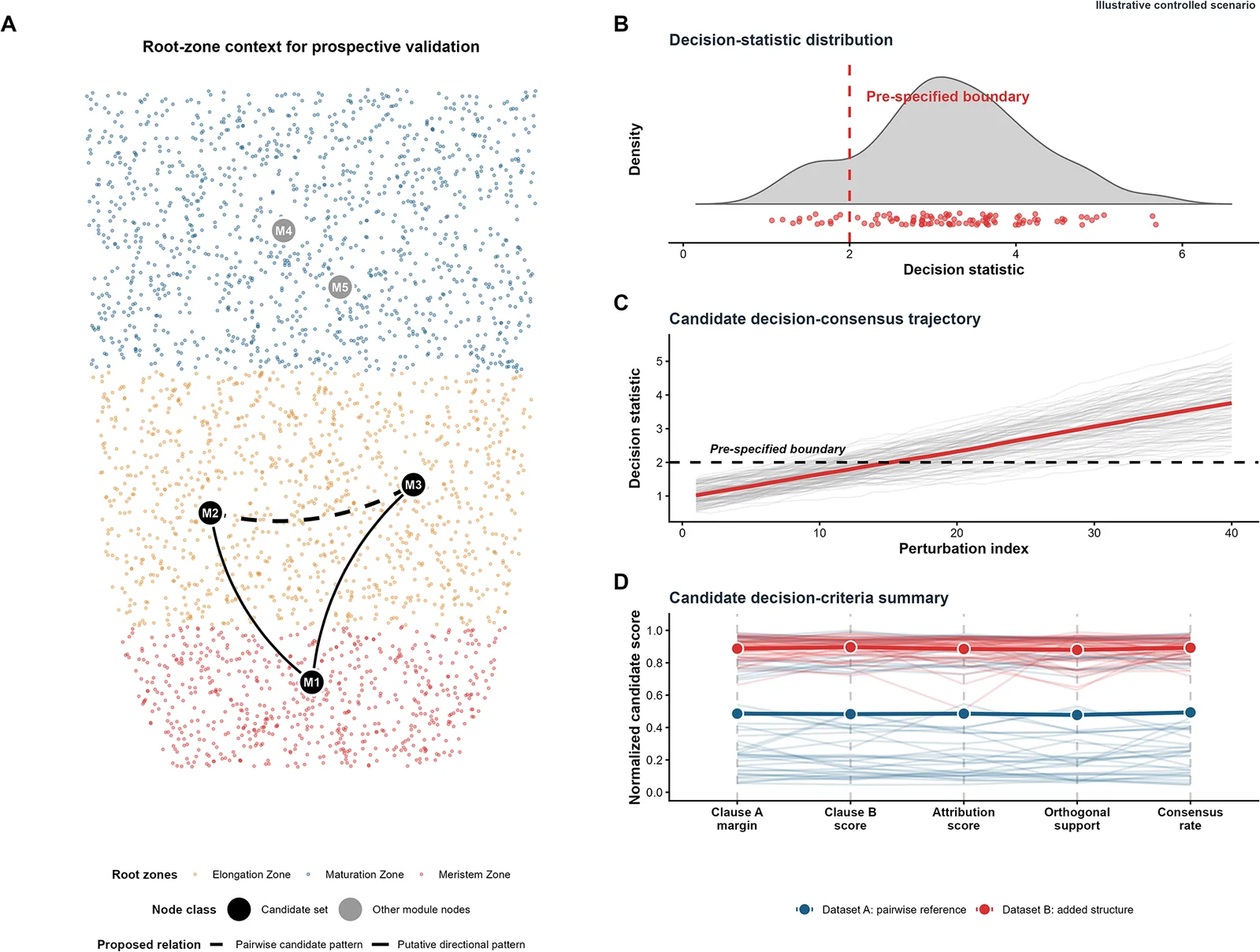
Prospective biological follow-up and measurement sensitivity readouts. The figure-level label identifies constructed displays for future biological follow-up and measurement sensitivity assessment. No empirical transcriptomic model was fitted. **(A)** A root point cloud separated into meristem, elongation, and maturation zones, with hypothetical module labels and non-causal relations. **(B)** Illustrative decision-statistic distribution. **(C)** Illustrative consensus trajectory under the proposed measurement sensitivity rule. **(D)** Parallel coordinate summary of the pairwise reference and added third-order scenarios using the Clause A margin, Clause B score, attribution score, orthogonal support, and consensus ratio. Panels **(B, C)** use constructed quantities, without raw trait error propagation or an empirical robustness estimate.

The prospective hypothesis is that the specified first-order plus pairwise predictor is non-inferior to the declared higher-capacity model under the chosen fitting and evaluation procedure. Clause Zero defines the study design, and phenotype calibration bounds physiological interpretation. A practically meaningful higher-capacity advantage would reject adequacy of the pairwise fit. A non-significant difference alone would not support it: the one-sided interval criterion in **Supplementary Methods S7** must be met.

## 2 From drought exposure to a physically interpretable root architectural target

### 2.1 A fixed four-trait scaffold for root architectural change

High-throughput imaging offers a growing range of traits for plant prediction (Wang et al., 2026a). Root phenotyping platforms can measure hundreds of variables (Paez-Garcia et al., 2015; Richard et al., 2015). This breadth makes the choice of an architectural target important before transcriptomic analysis begins. We therefore fix the trait combination before model fitting to avoid selecting a target after inspection of the results (Kerr, 1998). The framework therefore defines the RAD from a fixed four-trait matrix: total root length (TRL, *L*), root surface area (RSurfA, *S*), root volume (RV, *V*), and mean root diameter (RDmean, *D*).

The length and surface area describe soil exploration and root–soil contact, while the volume and diameter describe tissue allocation and radial construction. These coordinates are intentionally coarse. Anatomy and morphology also affect plant water status and drought tolerance (Zahedi et al., 2025). The framework captures macroscopic change without replacing those richer root traits (Richard et al., 2015; Lynch, 2022).

Several root phenes can change these same macroscopic coordinates. In maize, greater cortical aerenchyma reduced respiratory cost and was associated with greater root length density at depth, improved water status, and higher yield under water stress (Zhu et al., 2010). Reduced lateral branching also lowered respiration, increased deep-water capture, and improved the biomass and yield in mesocosm and field drought experiments (Zhan et al., 2015). These examples link root deployment and construction cost to drought performance. They also explain why a four-trait coordinate should complement, rather than stand in for, anatomy or branching topology.

We define the target as the one-dimensional generalized contrastive principal component ( ${\text{RAD}}_{\text{gcPC}1}$) of the fixed four-trait matrix. In Equation 1, *L*, *S*, *V*, and *D* denote the pooled-standardized TRL, RSurfA, RV, and RDmean, respectively, with loading weights *w*_1_–*w*_4_. **Supplementary Table S4** distinguishes this prospective scaffold from the inputs available in each public data branch.

(1) $$Y_{\text{RAD}}\equiv {\text{RAD}}_{\text{gcPC}1}=w_{1}L+w_{2}S+w_{3}V+w_{4}D$$

Equation 1 defines a candidate axis of relative drought-associated variation. Movement along it alone does not establish a mean treatment effect or a dose response. Classical contrastive principal component analysis (cPCA) maximizes foreground variance minus weighted background variance, making the direction dependent on the contrast weight *γ* (Abid et al., 2018). We instead use the generalized contrastive principal component analysis (gcPCA) variance ratio objective, which is fixed before transcriptomic fitting (de Oliveira et al., 2025). Its loading vector is the dominant generalized eigenvector.

(2) $$C_{fg}w=\lambda (C_{bg}+rI_{4})w$$

In Equation 2, C_fg_ and C_bg_ are the drought and well-watered covariance matrices, respectively; *w* is the four-component loading vector; and *λ* is the generalized eigenvalue. The fixed ridge is *r*, and I_4_ is the identity matrix. Both covariances use the same pooled training means and sample standard deviations (SDs). The gcPCA rule maximizes the drought-to-control variance ratio after adding an isotropic ridge to the background covariance. It removes the cPCA contrast-weight choice, but still depends on the specified ridge, scaling, and samples. The quotient describes variance, not the mean treatment response or physiological calibration. A leading eigenvalue at or below 1 indicates no foreground enrichment under this quotient: it does not automatically stop the analysis. **Supplementary Methods S1** gives the regularization and sign conventions (de Oliveira et al., 2025).

Clause Zero separates design admissibility from phenotype calibration. Independent soil water potential measurements record the exposure of each experimental unit, which may differ among genotypes receiving the same nominal treatment (Verslues et al., 2006; Chaves et al., 2002; Juenger and Verslues, 2023). The drying regime, sampling window, and measurement rules are set before inspecting the RAD associations or predictions. Calibration then estimates the direction, strength, uncertainty, and heterogeneity of the association between RAD and drought intensity, −Ψ_soil_, within genotypes and blocks. No correlation cutoff determines data inclusion. Weak, absent, or inconsistent calibration remains a reported outcome and limits physiological interpretation. **Supplementary Methods S1.2** specifies the prospective procedure.

The public data analyses compare geometric extraction objectives and their sensitivity to sampling. Independent calibration under Clause Zero is needed to give those coordinates a physiological interpretation.

### 2.2 Separating drought-enriched geometry from manual contrast choice

We used a public dataset of 12 *Aegilops*/*Triticum* species, comprising 720 observations with 360 per treatment (Pour-Aboughadareh and Poczai, 2021a). The archived 720-record PCA/cPCA branch retains its original one-decimal half-up preprocessing. The species screen and the corrected *Aegilops tauschii* record-level comparison use source precision, as described below. A separate durum experiment provides an external biological reference. Neither dataset records the independent water potential measurements required to validate RAD physiologically or to test the transcriptomic hypothesis.

#### 2.2.1 Global phenotypic variation contains a treatment-associated pattern

We first fitted an unsupervised PCA to all 20 physiological and root architectural traits (Lever et al., 2017). PC1 explained 32.4% of the total variance, and its scores separated the recorded treatment cohorts (**Figure 1A**). This shows a treatment-associated multivariate pattern in the observed data. Because the analysis includes physiological traits, it neither isolates the fixed four-trait architecture nor supplies an independent drought–response axis.

#### 2.2.2 Global axes mix root size with whole-plant physiology

The loadings show why global PCA is unsuitable as the RAD target (**Figure 1B**). The source-workbook fields RA, RSA, and NRL (retained under their original labels; **Supplementary Table S4**) contributed strongly to PC1, which mainly tracked the root system size. Root fresh weight, root dry weight (RDW), and the fresh weight root-to-shoot ratio contributed chiefly to PC2, connecting that axis with biomass and physiology. We therefore use the 20-trait components as descriptive references, not independent validation axes or components of the four-trait variance decomposition.

#### 2.2.3 Manual cPCA produces contrast-dependent root directions

Classical cPCA served as a parameter-sensitivity diagnostic (Abid et al., 2018). At *γ* = 0.70, the cPC1 loadings were RSA = 0.646, RV = −0.715, RD = 0.269, and TRLcalc = 0.003 (**Figure 2A**; **Supplementary Table S2**). For this archived 720-record branch, the restricted comparison retains only the RSA, RV, and RD from the global PCA and four-trait cPCA vectors. The non-equivalent NRL and TRLcalc coordinates are excluded. Both vectors use the same pooled-SD metric and are normalized to unit Euclidean length before calculating their absolute inner product. The absolute cosine with the restricted global PC1 was 0.053 (**Figure 2B**; **Supplementary Table S1**). Because the restricted references are non-orthogonal, this measures descriptive alignment, not an independent contribution or biological specificity. The *γ* = 0.70 result represents one manual setting rather than a validated drought coordinate.

#### 2.2.4 A modest change in *γ* shifts the apparent biological alignment

At *γ* = 0.50, the absolute cosines were 0.854 with the restricted global PC2 and 0.550 with the restricted global PC1 (**Figure 2B**; **Supplementary Table S1**). The change reflects sensitivity to the contrast parameter, not separation into independent biological dimensions. **Supplementary Figure S1A** similarly reports the observation-level standardized mean differences (SMDs) in scores. These differences do not establish independent experimental units, a measured drought gradient, or a RAD phenotype. Changing *γ* by 0.2 therefore changes the geometric comparison without supplying validation.

#### 2.2.5 The loading direction converges on global size variation as gamma decreases

Alignment with the restricted global PC1 increased from 0.053 at *γ* = 0.70 to 0.550 at *γ* = 0.50. It reached 0.808 at *γ* = 0.20, 0.939 at *γ* = 0.10, and 0.952 at *γ* = 0.05 (**Figures 2B, C**; **Supplementary Table S1**). The absolute cosines are non-additive because the restricted PC1 and PC2 references are non-orthogonal. **Figure 2B** plots the alignments separately, while **Figure 2C** presents the same values in a *γ*-by-reference matrix, with one cosine per cell. Thus, the cPCA family does not provide a RAD direction that is invariant to its contrast parameter.

This sensitivity matters when defining the phenotype before transcriptomic modeling. We chose gcPCA because its relative variance objective matches the intended target without requiring a cPCA contrast weight. That rationale selects an objective. It does not replace assessment of empirical stability.

#### 2.2.6 Restricting the genetic background does not remove manual parameter dependence

The full multi-species record-level analysis had no positive additive contrast direction at *γ* = 1. We therefore replaced the inadequately defined restricted subset with a complete-cohort screen and selected *A. tauschii* for the geometric illustration. All 12 source-defined species were screened using inclusive accession ranges, both treatments and both recorded replicates. The primary quality control (QC) unit was the accession-by-treatment mean. Within each species, pooled standardization and cPCA covered 39 gamma values from 0.05 to 1.00 in steps of 0.025, with 2,000 accession cluster bootstrap resamples. Eligibility and ranking combined cohort size, contrast feasibility, cross-gamma agreement, bootstrap reproducibility, and replication-specific feasibility and agreement (**Supplementary Methods S1.5**; **Supplementary Figure S2**; **Supplementary Table S5**).

The pooled multi-species accession mean analysis had positive leading contrasts at 21/39 gamma settings and a non-positive leading eigenvalue at *γ* = 1 (−0.000665). The complete *Triticum aestivum* cohort (19 accessions, 76 records) was feasible at 30/39 settings, but remained non-positive at *γ* = 1 (−0.0000411). Its bootstrap positive fraction was 53.55%. The screen selected *A. tauschii* (accessions 129–148; 20 accessions, 80 records): all 39 settings were feasible, the *γ* = 1 eigenvalue was 0.0134954, and the gamma 0.70-to-1.00 loading cosine was 0.990225. At *γ* = 1, 97.65% of the bootstrap fits were positive, with median absolute loading cosine of 0.990807 and 2.5th percentile of 0.441219. Both replication-specific fits were positive, with an inter-replication cosine of 0.650292 (**Supplementary Figures S2A–C**; **Supplementary Table S5**). These are species-level geometric QC results, not validation of plasticity, RAD, Clause A, or Clause B.

The record-level comparison in **Figures 3A, B** uses all 80 *A. tauschii* records from accessions 129–148, including 40 per treatment and both replications. Stored inputs are used without additional rounding. TRLcalc remains the operational product specific root length (SRL) × RDW, with unresolved physical units and calibration. RSA and RD retain their source field identities. Removal of additional rounding applies to every record. The accession-by-treatment mean QC already used source precision and is unchanged.

#### 2.2.7 gcPCA fixes the candidate direction without selecting gamma

We fitted the gcPCA and cPCA to the same pooled-standardized *A. tauschii* matrices. gcPCA used the dominant generalized eigenvector with absolute ridge 10^−6^, and cPCA used the original gamma registry (de Oliveira et al., 2025).

At source precision, the leading cPCA eigenvalue at *γ* = 1 was 0.02197533, while the gcPCA generalized eigenvalue was 1.898843. Their absolute directional cosine was 0.688172 (**Figures 3A, B**; **Supplementary Table S3**), replacing the earlier near-identity result. The eigenvalues represent different objectives and cannot be compared as performance measures. The corrected *A. tauschii* response was still sensitive to sampling. Across-replication correlation was negative, and the complete-refit mean response interval spanned zero (**Supplementary Table S7**).

The independent durum experiment comprised 36 pots from six genotypes, with direct measurements of root length (TRL), surface area (RSurfA), volume (RV), and mean diameter (RDmean) (Urbanavičiūtė et al., 2022a). The surface area and volume decreased in all six genotypes, providing clear evidence of an architectural treatment response. At *γ* = 1, the absolute cPCA-to-gcPCA loading cosine was 0.639882 (**Figures 3C, D**; **Supplementary Table S6J**).

In durum, gcPCA retained the training response sign in all six genotype holdouts, with a maximum leave-one-genotype-out rotation of 1.11°. The frozen cPCA comparator at *γ* = 0.7 gave four agreeing holdouts and 35.91°. These local diagnostics favor gcPCA under the specified extraction objective. **Figure 3** presents the separate comparison of *γ* = 1 loadings. Complete refitting perturbs the sample composition and re-estimates the scaling, covariances, and the axis. Under this broader perturbation, the response intervals for both methods spanned zero (**Supplementary Table S6B**). **Supplementary Figures S3A, B** report the gcPCA loading uncertainty and the fixed-axis *versus* complete-refit response intervals. Resistance to individual exclusions therefore coexists with uncertainty when the full coordinate is re-estimated.

#### 2.2.8 Geometric comparisons motivate independent physiological calibration

For comparison with the frozen global PCA reference, we mapped local coefficients into global pooled-SD coordinates. We then retained the RSA, RV, and RD and normalized the vectors to unit length, excluding the non-equivalent NRL and TRLcalc coordinates. The gcPCA absolute cosines with the restricted global PC1 and PC2 were 0.157117 and 0.191645, respectively. The *γ* = 1 cPCA values were 0.635219 and 0.795854, respectively. These separate, non-additive similarities use the archived global reference, not a source-precision global PCA rerun (**Supplementary Figure S1B**; **Supplementary Table S1**). Clause Zero addresses the next question: how do such geometric coordinates relate to independently measured physiological exposure?

### 2.3 Physical and physiological conditions required to call RAD plasticity

A physiological interpretation of root geometry requires both the exposure and the sampling phase to be defined. Gradual drying permits the study of growth adjustment before acute shock or terminal damage dominates. Phenotyping and transcriptome sampling within that window can then relate the root architecture to the molecular state (Verslues et al., 2006; McDowell et al., 2008; Juenger and Verslues, 2023).

First, the experiment requires a slow-drying design. In Equation 3, Ψ_soil_ denotes the soil water potential, *t* denotes time, and Δ denotes the change over the specified interval. The absolute drying rate must remain below an experiment-specific threshold for non-plastic osmotic shock ( $\kappa_{\text{shock}}$). Independent physiological evidence must justify that limit for the species, developmental stage, substrate, and measurement method without using the observed RAD associations.

(3) $$|\frac{\Delta \Psi_{\text{soil}}}{\Delta \text{t}}|\leq \kappa_{\text{shock}}$$

Second, phenotyping and transcriptome sampling must occur while the roots can still adjust their architecture. In Equation 4, Ψ_root_(*t*) is the root water potential at sampling time *t*. Ψ_stomata-close_ is the calibrated stomatal closure boundary, and Ψ_root-TLP_ is the root turgor loss point. Stomatal closure is operationally defined by the second-derivative zero of a locally weighted scatterplot smoothing with generalized cross-validation (LOESS-GCV) fit to stomatal conductance. The fitting domain and the treatment of multiple or absent inflections must be set in advance. The pressure–volume procedure and the window estimates also require independent physiological calibration or training data, without reference to the RAD associations or held-out results:

(4) $$\Psi_{\text{root}-\text{TLP}}\leq \Psi_{\text{root}}(t)\leq \Psi_{\text{stomata}-\text{close}}$$

The drying rate and sampling window constraints define the period intended for architectural adjustment before acute shock or terminal collapse (Koevoets et al., 2016; Lynch, 2022). They provide a context for water availability acclimation. They do not directly measure root hydraulics (Maurel and Nacry, 2020) or establish geometric stability.

Clause Zero defines the experimental conditions before outcome inspection and reports the physiological calibration separately. Dataset inclusion, exclusions, and sampling windows cannot be changed after examining the RAD associations. Monotonicity must be assessed within genotypes and blocks along the measured water status trajectory since a pooled association may conceal confounding. Calibration failure does not halt or retune the pre-specified Clauses A and B for a defined RAD target. It limits their interpretation to the prediction of the geometric coordinate rather than the calibrated drought plasticity (**Supplementary Methods S1.2**).

## 3 From comparable root transcriptomic states to a prospective test of pairwise adequacy

Having defined the root coordinate and its physiological context, we turn to transcriptomic organization. Bulk root transcripts are strongly dependent because genes share regulation and samples differ in tissue composition. Co-expression modules offer a lower-dimensional description of these coordinated states. We ask whether individual module states and two-module configurations contain enough information to represent RAD. Relative topology deviation informs continuous penalties before that test, without automatic exclusion. Collinearity within the resulting predictor space must then be addressed separately.

### 3.1 Co-expression modules as cross-treatment state variables

Drought-responsive transcripts often vary together, which can make gene-level predictors unstable and difficult to relate to root biology. Weighted co-expression analysis summarizes these correlations as modules without requiring prior pathway annotation (Zhang and Horvath, 2005; Langfelder and Horvath, 2008). Their eigengenes provide compact descriptions of the transcriptomic state that can be related to RAD.

A co-expression module summarizes coordinated expression: it is not itself an organ or pathway. Fixed reference membership and loadings provide common coordinates across treatments, avoiding independently fitted axes. Shared coordinates nevertheless do not establish a common mechanism or biological identity (Yip and Horvath, 2007). Remodeled reference modules remain in the continuously weighted basis. Drought-emergent modules and recombined signals absent from that basis may be missed. Conclusions are therefore restricted to the predefined predictors, with anatomy, physiology, and perturbation needed to connect them to root processes (Barabási and Oltvai, 2004; Civelek and Lusis, 2014).

Cereal transcriptomes illustrate this dependence on a biological context. Rice weighted gene co-expression network analysis (WGCNA) studies identified modules and hub genes in the roots, leaves, and panicles. In barley seminal roots, modules differed among the root cap, meristem, and the elongation and differentiation zones under water deficiency (Smita et al., 2013; Klaus et al., 2024). These findings support context-specific expression states, but do not make an eigengene product a physical interaction or pathway.

### 3.2 Assess relative topology deviation before weighting joint states

Reference eigengenes provide common coordinates for comparing module states. Because shared coordinates do not ensure biological comparability, changes in the co-expression topology inform the proposed continuous penalties. In Equation 5, the relative topology deviation $\Delta R_{i}$ compares the drought and reference TOM sub-matrices on the same genes using the Frobenius norm ||·||*F*. *T_i,_*_fg_ and *T_i_*_,bg_ denote the matched foreground and background TOM blocks of module *i*, respectively.

(5) $$\Delta R_{i}=\frac{∥T_{i,\text{fg}}-T_{i,\text{bg}}{∥}_{F}}{\text{max}(10^{-6},\;∥T_{i,\text{bg}}{∥}_{F})}$$

ΔR expresses the topology change relative to the estimated reference and is dimensionless. Smaller values indicate smaller relative changes, not established statistical stability, module preservation, or biological identity. Normalization does not ensure invariance to module size. Sample size and network/TOM choices also matter. **Supplementary Methods S2** specifies the denominator safeguard and the empirical calibration needed to interpret the score (Zhang and Horvath, 2005).

In the prospective protocol, well-watered reference samples within each training level define gene membership, preprocessing, eigengene loadings, and orientation. The stored transformation is applied to the drought, validation, and test samples. TOM estimation is performed separately by condition on matched gene sets. In contrast, the controlled implementation estimates eigengenes directly from foreground expression and does not perform reference projection (**Supplementary Methods S2.3**, **S2.4**). WGCNA preservation diagnostics are a complementary comparison for future empirical work (Langfelder et al., 2011).

The controlled co-expression scenarios contain three module states with different relative deviations: M1 ( ΔR=0.0462), M6 ( ΔR=1.9474), and M9 ( ΔR=0.7528). Their penalties illustrate how the weighting changes with reorganization. **Figures 4A–C** do not define an admissibility cutoff, rank biological relevance, or calibrate sampling noise, but represent controlled examples rather than a measured drought network.

### 3.3 Formalization of pairwise representation prior to model fitting

Clause B asks whether the specified first-order plus pairwise predictor is adequate relative to a pre-specified higher-capacity model under the chosen fitting procedure. Correlated eigengenes can produce misleading candidate products; therefore, the credibility of a particular candidate is assessed separately from the overall predictive adequacy (Tibshirani, 1996; Heinze et al., 2018).

The first-order plus pairwise Hadamard predictor basis is defined before fitting:

(6) $$X=[{\left\{M_{i}\right\}}_{i=1}^{K}\cup {\left\{M_{i}\circ M_{j}\right\}}_{1\leq i<j\leq K}]$$

For Clause B, *K* is the number of base modules in U_B. The pre-specified transcriptome QC and reference module rules define U_B within the training data without inspecting the outcome. Each eigengene M_i is centered and divided by its training sample SD before constructing *i* < *j* products. The stored transformations are then applied to the validation and test samples. Equation 6 contains *K* main effects and *K*(*K* − 1)/2 unordered products, without squares or duplicates. The prospective pairwise model retains every base main effect term without penalization and sparsely penalizes only products. This is hierarchical term inclusion, not a guarantee of non-zero main effect coefficients; ordinary LASSO does not enforce that guarantee. **Supplementary Methods S7** specifies the fitting feasibility and training isolation.

Water deficit responses can differ sharply among root tissues and developmental zones. In maize primary roots, fewer than 3% of responsive genes changed across all sampled tissues, whereas more than 75% responded in only one. Cortex and elongation zone samples showed extensive responses, including cell wall reorganization near the root tip (Opitz et al., 2016). A module product could therefore reflect tissue composition or a zone-specific program. Topology weighting favors a particular co-expression structure: it neither corrects cell mixture confounding nor establishes a shared cellular program. A remodeled module may still be biologically important.

Inference about interaction order requires explicitly defined function classes (Phillips, 2008; Mackay, 2014; Taylor and Ehrenreich, 2015). Equation 6 omits squares and nonlinear main effects. A higher-capacity model could improve by capturing these terms, thresholds, or localized responses or through its fitting procedure (Friedman, 2001; Elith et al., 2008). Such improvement would not identify higher-order module coordination. Nested polynomial or functional ANOVA comparisons could address that separate question in future work.

We propose a continuous topology-informed penalty prior (Equation 7) that favors products with smaller summed relative topology deviations. This preference retains the remodeled reference modules through continuous weighting rather than applying a new exclusion threshold.

(7) $$\Lambda_{i,j}=\frac{1}{1+\Delta R_{i}+\Delta R_{j}}$$

The mapping is bounded on (0,1], differentiable, decreasing, and convex in the non-negative summed deviation. It requires no fitted rate constant. Λ is a penalty prior, not a probability or an automatic module eligibility rule. The proposed rule uses expression data from the relevant training partition, without RAD-based selection (**Figures 5A, B**; **Supplementary Methods S3**).

The assigned penalty multiplier is 1.0723 for the coherent candidate label M1 × M2 and 2.6943 for M9 × M10. The correlated alternative M3 × M4 receives 1.0567. The low penalty on this alternative shows why topology weighting alone cannot distinguish coordination from correlation (**Figure 5B**). **Supplementary Methods S2.3** explains the archived eigengene label convention and its limitations.

### 3.4 Protect the pairwise screen from collinearity

Collinearity is a central difficulty in the pairwise space. Several products may predict the same response; thus, a selected term may be a proxy rather than a reproducible joint effect. Locked LASSO fitting and matched target-decoy screening help organize these competing candidates. Their purpose here is candidate triage within a specified analysis, not biological discovery.

We assess candidate retention within the specified analysis using locked LASSO fitting (Tibshirani, 1996; Meinshausen and Bühlmann, 2010). For both original predictors and their matched continuous decoys, the same topology-based penalty multiplier, the inverse of Λ*k*, is used for the augmented matrix [ X, $\tilde{X}$] in Equation 8. Each original predictor and its decoy therefore receive matched penalties. In Equation 8, *Y* is the RAD response for *n* observations. X and $\tilde{X}$ contain *p* original predictors and their matched decoys, with separate coefficient vectors ***β*** and $\tilde{\beta }$. The matched factor *p*_k_ is the topology-derived penalty multiplier for predictor *k*, and $\lambda_{1\text{se}}$ is the locked regularization strength. The augmented LASSO is fitted to *Y* in 100 subsamples, each containing 50% of the observations. This controlled screen differs from the prospective Clause B fit in **Supplementary Methods S7**. Its output does not establish biological interactions or hierarchical inclusion of terms.

**Supplementary Table S4** lists the operational constants, their scope, and the parameters that require experiment-specific values.

(8) $$\underset{\beta ,\;\tilde{\beta }}{\min}\{\frac{1}{2n}∥Y-X\beta -\tilde{X}\tilde{\beta }{∥}_{2}^{2}+\lambda_{1se}\overset{p}{\underset{k=1}{\sum }}p_{k}(|\beta_{k}|+|{\tilde{\beta }}_{k}|)\}$$

The controlled stability analysis uses the conservative 1 standard error value, $\lambda_{1\text{se}}=0.1509$, rather than the minimum error value, $\lambda_{\min}=0.0542$, to favor sparse, stable candidates over a tighter fit (**Figure 6A**). In the regularization path, majority of the correlated alternatives shrink to zero, while the pre-specified coherent candidate retains its coefficient (**Figure 6B**) (Friedman et al., 2010).

In this controlled draw, Ω*k* is the original feature retention frequency from the augmented target-plus-matched decoy fits (**Figures 6C, D**). The coherent candidate has Ω=0.93 at the operational stability reference Ω≥0.60, while the topology-downweighted candidate has Ω=0.24. The correlated M3×M4 term still falls below the stability reference under resampling ( Ω=0.57), showing why the two steps are used together. These frequencies describe operational stability under the locked- λ resampling protocol (**Supplementary Methods S4**).

The retention frequency Ω summarizes operational stability rather than biological discovery. Highly collinear terms such as M3×M4 can reach the reference without representing a reproducible joint state. Target-decoy screening therefore pairs each original feature with a decoy receiving the same topology penalty. The construction resembles knockoffs, but does not assume Model-X exchangeability or provide a false discovery guarantee (Barber and Candès, 2015; Candès et al., 2018).

The original and paired-decoy covariance matrices look similar in the configured draw; however, visual similarity does not establish exchangeability. We define *W*_k_=Ω_k_− $\tilde{\Omega }$_k_, where $\tilde{\Omega }$_k_ is the matched decoy retention frequency. At the display threshold *τ* = 0.910, the coherent feature enters the candidate region, while its decoy and correlated counterparts do not (**Figures 7A, B**). **Supplementary Methods S5** gives the construction, containment, and filtering rules.

At a minimum inclusion level of 25%, the no-penalty, unlocked cross-validation, and no-decoy approaches retain more correlated alternatives. The proportions of wrongly included terms range from 33.3% to 77.8%. In the same draw, topology-informed target-decoy screening retains only the pre-designated coherent term (**Figure 7C**). This comparison describes one controlled draw, not a formal false discovery rate (FDR) guarantee or an evaluation of the operating characteristics.

At *τ* = 0.550, the directly generated scores in **Figure 8** place 39 terms in the candidate region, 16 in the weak status region, and 2,445 in the null status region. These screening labels differ from the planted signal and correlated null classes used to construct the scenario. The 39 candidates proceed to Clause A only. They are not causal or FDR-controlled discoveries and do not restrict U_B or either Clause B comparator.

## 4 Decision readouts in constructed scenarios

The 39 nominal candidates from *Section 3* supply the Clause A illustration. Clause B uses the full pre-specified module universe even if no Clause A candidate survives. **Figure 8** and the following constructed scenarios distinguish these two questions without applying them to empirical drought transcriptomes (Nosek et al., 2018; Kerr, 1998).

### 4.1 Two scenarios with pre-specified signal structure

We illustrate the decision logic with controlled scenarios whose signal structure is specified in advance. Dataset A is a pairwise reference scenario containing the first- and second-order Hadamard structures, with no third-order signal ( $\eta_{3\text{rd}}=0$). Dataset B retains the first- and second-order structures, but includes a declared third-order functional term ( $\eta_{3\text{rd}}>0$).

Both scenarios have the same declared block-diagonal covariance structure, but with dataset B adding the specified third-order functional term. The errors and attributions in **Figure 9**, however, were generated directly rather than obtained by fitting models to those responses. They illustrate possible readouts, not recovered interaction order or measured predictive performance.

### 4.2 Clause A asks whether a candidate exceeds structured background correlation

Clause A asks whether a nominated term is retained more often than the strongest structured-null competitor within a pre-declared candidate family. The prospective test repeats every response-dependent selection step, including tuning and screening, for the observed RAD target and each permissible pseudo-response.

The prospective test adds fitted nuisance values back to permuted nuisance residuals. Permutation respects justified exchangeability blocks and keeps repeated observations together within biological units. Within each outer training partition, the observed response and every permissible pseudo-response undergo the same outcome-dependent preprocessing, tuning, screening, *λ* selection, and applicable *τ* or boundary selection. The predictor definitions and settings may remain fixed only if outcome-blind, externally pre-specified, or independently calibrated. Let $C_{b}$ be the response-specific nominated subset of the predeclared pair family $U_{A}$, with b=0 denoting the observed response (Winkler et al., 2014). The structured-null maximum is defined in Equation 9.

(9) $$M_{\text{null}}^{\left(b\right)}=\underset{k\in C_{b}}{\text{max}}\Omega_{k}^{\left(b\right)},\;C_{b}\subseteq U_{A}$$

**Supplementary Methods S6** specifies the nuisance model, permissible transformations, candidate family, and tie-inclusive upper tail. With the prospective budget of 1,000 permutations, a candidate must strictly exceed the 95% max-null ceiling. Interpretation depends on the experiment’s exchangeability and nuisance assumptions. The constructed display provides no empirical error-control result.

**Figure 9A** illustrates this question using constructed coefficient trajectories for noise and a coherent term. They are not fitted permutation results. The fixed-penalty display and the controlled locked-LASSO results show the intended readouts. The full-procedure test in **Supplementary Methods S6** remains prospective and separate from Clause B.

### 4.3 Clause B assesses predictive adequacy of the specified basis

Clause B compares the Equation 6 predictor with a pre-specified higher-capacity model on the same target, module universe U_B, and held-out units. Transcriptome QC and reference module rules define U_B without RAD association screening, Clause A selection, target-decoy *τ*, or retention frequency Ω. Module availability rules must be outcome-blind and identical for both models; continuous Δ*R* adds no exclusion threshold. Clause B proceeds even when Clause A has no survivors. The result concerns the specified basis and fitting procedure, not general interaction order (**Supplementary Methods S7**).

The capacity illustration starts from standardized base module eigengenes $\left\{M_{i}\right\}$. A single regression tree *T* with maximum depth *D* is represented by binary indicator functions I(·). At D=2, Equation 10 gives a restricted form whose paths use the same two module coordinates; a general depth 2 tree may use different second-split variables on different branches. Here, ***x*** is the vector of the standardized module scores, *m* indexes the four terminal regions, *c_m_* is the prediction in region *m*, and *R_m_*_1_ and *R_m_*_2_ are its split-defined intervals. *I* is one when its condition holds and zero otherwise. *D* denotes the tree depth in this local equation. These threshold indicator products are different from the Hadamard products in Equation 6.

(10) $$T(x;D=2)=\overset{4}{\underset{m=1}{\sum }}c_{m}I(M_{i}\in R_{m1})I(M_{j}\in R_{m2})$$

**Figure 9B** contains constructed surfaces, not fitted depth 2 or depth 3 trees. A deeper fitted model could improve by capturing the nonlinear main effects, thresholds, piecewise responses, or localized structure. Its advantage would not identify a three-module mechanism or all signal beyond pairwise interactions (Friedman, 2001; Elith et al., 2008).

**Figure 9C** shows generated errors rather than cross-validation estimates. For an empirical comparison, define Δ = RMSE_pairwise − RMSE_higher on the same standardized RAD scale; therefore, positive values favor higher capacity. The margin δ* must be justified from biological or predictive utility before evaluation. Technical replicate error informs precision and feasibility, not the margin. At one-sided *α* = 0.05, a lower bound above δ* supports a meaningful higher-capacity advantage, while an upper bound below δ* supports non-inferiority of the pairwise fit. Bounds touching or spanning δ* are indeterminate. Conditional test set simulations show scenario-dependent power (**Supplementary Figure S4A**) and elevated small-sample boundary rejection (**Supplementary Figure S4B**; **Supplementary Table S8**). They do not compare fitted empirical transcriptomic models.

**Figure 9D** assigns illustrative importance values to pre-labeled terms. These were not estimated from a model or Shapley additive explanations (SHAP) analysis. Following an empirical Clause B comparison, a declared attribution method could guide biological follow-up without establishing interaction order or causality (Lundberg et al., 2020).

### 4.4 Localization of remaining candidates in root tissue and developmental stages

Clause A candidates and hypotheses suggested by a future Clause B attribution analysis need biological follow-up. Localization can establish whether their parent modules occur in compatible tissues, cell types, or developmental stages. Causation requires perturbation. These follow-up candidates are not fed back into U_B.

**Figure 10A** illustrates possible spatial follow-up by placing hypothetical module labels on a simulated root point cloud containing n=3,000 cells. The illustrative manifold contains a meristem zone with 628 cells, an elongation zone with 1,122 cells, and a maturation zone with 1,250 cells. Spatial transcriptomics or anatomy could test whether candidates occupy such root contexts. The illustration does not show these module combinations occurring *in vivo* (**Figure 10A**) (Nobori et al., 2023).

Rice single-cell and spatial transcriptomics illustrate how this follow-up could work. Under soil compaction, stress-responsive programs were localized to vascular, exodermal, and endodermal cell types, including abscisic acid (ABA) signaling and lignin/suberin barrier programs. The same study measured barrier deposition and radial water loss (Zhu et al., 2025). Although compaction differs biologically from drought, the design links cell identity, wall remodeling, and hydraulic consequences. **Figure 10A** illustrates the prospective placement of module labels, not such measurements.

CARNIVAL offers a complementary method to organizing future candidates into plausible directed regulatory triplets (Liu et al., 2019). Agreement with a network model would strengthen a candidate’s rationale, without establishing causality. Spatial localization, independent prediction, and perturbation of the relevant genes or tissues would still be needed before identifying a drought response pathway.

### 4.5 Test whether image-derived trait uncertainty changes the decision

RAD is dependent on image-derived root length, surface area, volume, and diameter. Measurement variations can therefore affect module state tests (Richard et al., 2015). The values σ=0.05 and 100 perturbation runs are assumptions for the controlled display, not empirical settings or results. An empirical analysis first needs the joint error distribution of the four traits, estimated from repeat scans, segmentations, manual corrections, operators, or batches. Raw traits are then perturbed, followed by repeated standardization, RAD extraction, and the full decision procedure. This tests how trait measurement affects the RAD-dependent decision rather than establishing the robustness of a biological pathway. **Supplementary Methods S8** gives the Measurement-Fragile rule.

The measurement sensitivity display combines a decision-statistic distribution and a consensus trajectory (**Figures 10B, C**). Both are constructed scenario readouts, not *p*-values or robustness estimates from repeated root measurements. An empirical analysis would perturb the raw traits under the declared error model, re-extract RAD, and propagate uncertainty through the full decision procedure.

The agreement ratio serves as an operational warning. Consensus below 70% triggers the Measurement-Fragile label, while consensus at or above 70% means only that this warning was not triggered. The illustrative cutoff has no calibrated error control or robustness interpretation (**Supplementary Methods S8**).

### 4.6 Controlled scenario readout

**Figure 10D** brings together the constructed Clause A, Clause B, attribution, orthogonal support, and measurement consensus coordinates for both scenarios. It shows how future outcomes could be interpreted rather than decisions from an executed workflow. Biological follow-up then addresses candidate credibility and predictive basis adequacy as separate questions (**Supplementary Methods S9**).

## 5 Discussion

### 5.1 What biological question does the framework make testable?

We ask how much transcriptomic predictive complexity is needed for a fixed root architectural target. More specifically, is the first-order plus pairwise fit adequate relative to a pre-specified higher-capacity model? Physiological calibration determines how that target can be interpreted. The comparison can guide biological hypotheses, but does not identify drought sensing, tissue-to-tissue signaling, or cellular control of growth (Platt, 1964; Chambers and Tzavella, 2022).

### 5.2 What does Clause Zero establish about the RAD phenotype?

Clause Zero connects the coordinate to physiological exposure. Nominal treatment groups support comparisons of root traits, whereas the measured water potential locates each experimental unit along an exposure trajectory. Defining the drying regime and sampling window then relates that trajectory to root growth and transcriptomic sampling (Juenger and Verslues, 2023). Geometric stability asks whether the coordinate persists under specified perturbations. Calibration asks what exposure–response relationship it represents, a question that remains even for a stable direction.

The durum treatment response and favorable local gcPCA diagnostics support the extraction rule within the tested comparison. *A. tauschii* reveals greater sampling sensitivity. Together, these examples make the case for studying geometric stability and physiological exposure in the same experiment. Unrecorded variations in the stress intensity or the developmental stage could contribute to response uncertainty (Juenger and Verslues, 2023). Without water potential records, this explanation remains untested. The missing records do not show that either experiment had an unsuitable gradient. Matched phenotype–transcriptome measurements and independent water potential trajectories could resolve this question in held-out units while retaining the genotype and block structure.

A weak calibration result limits a drought plasticity interpretation, but does not erase a predictive result for the defined geometric target. Clauses A and B therefore retain their pre-specified rules. A new cohort, sampling window, or calibration specification would belong to a separately declared analysis rather than a revision made after inspecting the outcome.

### 5.3 How should candidate failure and predictive inadequacy be interpreted?

Both failed candidates and unfavorable predictive comparisons are informative. Clause A concerns credibility of an individual term. A higher-capacity gain beyond the utility margin instead concerns inadequacy of the specified pairwise fit, regardless of candidate survival (Ioannidis, 2005; Chambers and Tzavella, 2022). Both outcomes need to be reported.

Practical adequacy requires the one-sided upper 95% bound for Δ to fall below δ*. This is non-inferiority to the declared comparator, not two-sided equivalence. An imprecise interval or non-significant contrast is insufficient. The conclusion applies to the chosen target, module universe, fitted models, and evaluation population.

A failed Clause A candidate does not determine the Clause B result. Conversely, a meaningful higher-capacity advantage identifies a limitation of the pairwise fit, potentially involving nonlinear main effects or other omitted structure. Neither result establishes a three-module process or a lower bound on the biological interaction order.

### 5.4 How should the boundary be tested prospectively?

Prospective evaluation requires the choices in *Section 4* to be registered before outcomes are inspected. These include the phenotype, Clause Zero measurements, module matching, topology, resampling, penalties, decoy construction, and decision criteria. Standardization, gcPCA loadings, and data-dependent sign or calibration choices are fitted within the training data or fixed by independent calibration. Held-out RAD scores use these stored quantities without refitting. Held-out data do not select directions, thresholds, or windows. Clause A repeats response-dependent tuning and screening under each permissible residual permutation (**Supplementary Methods S6**). Clause B gives both models the same U_B and held-out units. **Supplementary Methods S7** evaluates non-inferiority conditionally for frozen models on an independent test set, without counting overlapping cross-validation folds as independent replicates. Applying the design analysis in **Supplementary Methods S7.5** to biology still requires experiment-specific utility, variance, and sampling assumptions.

The present evidence serves three purposes. Public phenotypes reveal treatment responses and sampling uncertainty; controlled illustrations define the intended readouts; and independent test simulations examine conditional power and interval behavior. The small-sample limitations in **Supplementary Methods S7.5** are part of these findings. Empirical pairwise adequacy and the operating characteristics of the complete workflow remain to be tested.

The sequence from RAD geometry to biological follow-up organizes these questions without making Clause B dependent on candidate survival. Keeping calibration, candidate credibility, and predictive adequacy separate allows each result to retain its own interpretation. Training isolation protects these distinctions during evaluation (Nosek et al., 2018; Chambers and Tzavella, 2022).

### 5.5 What evidence is needed to move from prediction to mechanism?

Non-inferiority would support use of the pairwise fit within δ* for the declared comparator and population. It would not establish that all higher-order information is absent or that biological coordination occurs only in pairs. Those claims require richer, explicitly defined function classes and independent biological evidence.

A meaningful higher-capacity gain would instead identify predictive structure missing from the pairwise fitting procedure for the evaluated target and population. It would not identify the biological order or mechanism of that structure. Follow-up would compare explicit richer representations and perturb candidate genes, modules, tissues, or developmental processes. Model depth alone cannot answer those biological questions.

Root physiology suggests several possible sources of that predictive structure. Growth angle and rooting depth affect drought tolerance in rice (Uga et al., 2013). Cortical construction cost and lateral branching trade-offs influence deep-water capture in maize (Zhu et al., 2010; Zhan et al., 2015). Expression responses also vary among root tissues and developmental stages (Opitz et al., 2016; Klaus et al., 2024). Distinguishing these explanations requires physiological calibration, independent prediction, cell type localization, and perturbation, followed by genotype × environment and yield tests.

## 6 Conclusion

The framework poses a pre-specified prediction question about root architectural change and transcriptomic coordination. RAD geometry and Clause Zero define the target and its physiological context. Module continuity and the first-order plus pairwise Hadamard basis define the predictors. Clause A assesses individual candidates, while Clause B evaluates predictive adequacy over the full outcome-blind module universe (Nosek et al., 2018; Chambers and Tzavella, 2022).

Public wheat analyses contribute treatment response evidence while exposing source precision and complete-refit stability limitations. Controlled illustrations show intended readouts, and the conditional design study informs test set planning under specified error assumptions. Whether the pairwise fit is practically adequate remains a prospective question under Clause Zero, assessed against a utility-based δ* using one-sided non-inferiority.

A non-inferiority result would support retaining the pairwise fit for its declared use, while a meaningful deficit would motivate richer predictors. Either result would guide physiological and mechanistic follow-up, without determining general biological interaction order (Nosek et al., 2018; Chambers and Tzavella, 2022).

## Data Availability

The wheat phenotyping data are available in Mendeley Data as “A dataset on multi-trait selection approaches for screening desirable wild relatives of wheat,” Version 1 (Pour-Aboughadareh and Poczai, 2021b), doi: 10.17632/84kmmmgvvr.1. The independent durum wheat root measurements associated with Urbanavičiūtė et al. (2022a) are available in Zenodo, version 1.0 (Urbanavičiūtė et al., 2022b), doi: 10.5281/zenodo.5883299. Both source datasets use CC BY 4.0 licences. The earlier numerical outputs and scientific source tables, including the fixed-seed controlled computational scenarios, are archived in Zenodo version 1.0.0 at https://doi.org/10.5281/zenodo.21890013 under CC BY 4.0. The controlled outputs are computational scenarios rather than experimental observations. This archive version does not contain the source-precision and external-reference updates in the present revision. The revised numerical data are publicly available in Zenodo version 1.1.0 at https://doi.org/10.5281/zenodo.22653370 and in GitHub release v1.1.0 at https://github.com/ShaunAsherLeo-oss/rad-interaction-order-framework/releases/tag/v1.1.0, under CC BY 4.0. The DATA archive contains the complete 12-species QC, source-precision A. tauschii response and stability diagnostics, the independent durum reference, the conditional test-set design simulation, and scientific source tables for **Figures 1**–**10** and **Supplementary Figures S1**–**S4**. A figure-to-source index, data dictionaries, checksums and the complete revised Supplement accompany the release. Historical and current analytical branches are identified separately. The source-faithful numerical R implementation is available from GitHub at https://github.com/ShaunAsherLeo-oss/rad-interaction-order-framework (release v1.1.0; commit 98481cc362f64d09276dd663851e8fc13ac9c7ea; MIT License) and archived in Zenodo Version 1.1.0 at https://doi.org/10.5281/zenodo.22653276. The release includes versioned configuration, deterministic analysis drivers, numerical source-table builders, regression tests and an R package/environment lock. It reproduces the implemented descriptive analyses and controlled computations and does not implement the prospective full-procedure Clause A test in **Supplementary Methods S6** or the reference eigengene projection in **Supplementary Methods S2.4**. The figures were prepared in R 4.3.3. Complete panel-level numerical source tables, the figure-to-source index and software versions support independent inspection and replotting. The earlier compute-only implementation remains archived at https://doi.org/10.5281/zenodo.21890088 (version 1.0.0).
