# Supplementary material for: A prospective framework for testing whether pairwise transcriptomic module coordination can represent drought-associated root architectural change
Source: Front Plant Sci. 2026 Oct 16;17:1965677. doi: 10.3389/fpls.2026.1965677 (PMC13719389; doi:10.3389/fpls.2026.1965677)
Supplement: Supplementary file 1 [file SupplementaryFile1.docx]

Supplementary Material

# 1 **Supplementary Methods**

These supplementary methods distinguish the implemented descriptive and controlled analyses from the prospective procedures of the framework. S7.5 reports the separate conditional independent-test simulation study for frozen predictors. The complete empirical workflow remains prospective. Co-expression modules, pairwise products, and higher-capacity probes are transcriptomic state variables and statistical test objects rather than direct evidence of causal drought-response pathways.

## **S1. Construction of the generalized contrastive RAD target using gcPCA**

The gcPCA extraction rule targets the registered foreground-to-background variance ratio before transcriptomic modelling. It avoids selection of a cPCA contrast weight while retaining explicit choices of ridge, scaling and sample definition. The mathematical rule and empirical stability of a fitted direction are assessed separately.

Let $\text{X}_{\text{fg}}\text{∈}\text{ℝ}^{\text{n}\text{×4}}$ and $\text{X}_{\text{bg}}\text{∈}\text{ℝ}^{\text{m}\text{×4}}$ denote the foreground and background phenotypic matrices, respectively. The foreground matrix corresponds to drought-treated plants, and the background matrix corresponds to well-watered plants. The prospective columns are total root length (TRL), root surface area (RSurfA), root volume (RV) and mean root diameter (RDmean), corresponding to the standardized L/S/V/D coordinates in Equation 1. Table S4 separately maps the actual inputs of each public-data branch. Trait values are column-centered and scaled before covariance estimation. Their empirical covariance matrices are denoted by $\text{C}_{\text{fg}}$ and $\text{C}_{\text{bg}}$.

The objective of gcPCA is to identify a projection vector $\text{w}$ that maximizes the variance of the foreground phenotypic space relative to the background phenotypic space. Before covariance matrices are estimated, the drought and well-watered four-trait matrices are centered and scaled once with pooled means and standard deviations computed across both groups. The identical fitted transformation is applied to both groups, so the covariance quotient is evaluated in one common feature coordinate system. This is formulated as the generalized Rayleigh quotient.

$$\max_{\text{w}}\frac{\text{w}^{\text{T}}\text{C}_{\text{fg}}\text{w}}{\text{w}^{\text{T}}\text{C}_{\text{bg}}\text{w}}\text{ }\text{ }\text{ (}\text{Equation S1}\text{)}$$

Stationarity of this quotient gives the generalized eigenvalue problem.

$$\text{C}_{\text{fg}}\text{w}\text{=}\text{λ}\text{C}_{\text{bg}}\text{w}\text{ (}\text{Equation S}\text{2}\text{)}$$

The RAD loading vector $\text{w}_{\text{RAD}}$ is defined as the generalized eigenvector associated with the largest generalized eigenvalue $\text{λ}_{\text{max}}$. This extraction rule is fixed before downstream modeling and does not require a manually chosen contrast parameter. For any phenotypic sample matrix $\text{X}$ aligned to the transcriptomic samples, the RAD score is computed as follows.

$$\text{Y}_{\text{RAD}}\text{=}\text{X}\text{w}_{\text{RAD}}\text{ (}\text{Equation S}\text{3}\text{)}$$

Both treatments are projected onto the same fitted axis in each public-data comparison. Treatment contrasts describe the architectural response, exclusion fits assess local influence, and complete-refit resampling evaluates uncertainty when the coordinate is re-estimated. Physiological calibration then relates the coordinate to independently measured water status (S1.2).

## S1.1 Numerical stabilization for near-singular background covariance matrices

In genetically homogeneous or low-variance experimental settings, $\text{C}_{\text{bg}}$ may be ill-conditioned. After the common pooled standardization described above, a pre-specified ridge stabilization term is applied only for estimation of the projection vector. This ridge does not select a contrast penalty and is not optimized against RAD separation, transcriptomic prediction, or any downstream outcome.

$$\text{C}_{\text{bg}\text{,ϵ}}\text{=}\text{C}_{\text{bg}}\text{+ϵ}\text{I}_{\text{4}}\text{ (}\text{Equation S}\text{4}\text{)}$$

The public single-species analyses use fixed absolute ridge r = ϵ = 10⁻⁶ after common pooled standardization. I₄ in Equation S4 is the four-dimensional identity matrix. A positive ridge adds a common amount to the background eigenvalues, keeping the denominator defined along directions with little background variation. The chosen magnitude is a numerical reference for comparing fits, with scientific sensitivity evaluated over the complete frozen relative-ridge grids. Background condition numbers change only modestly at this reference value (Tables S6b and S7; accompanying baseline_geometry.csv files). This motivates reporting the spectrum, bootstrap uncertainty and the entire ridge response, rather than inferring stability from solvability. The regularized eigenproblem is:

$$\text{C}_{\text{fg}}\text{w}\text{=}\text{λ}\text{C}_{\text{bg}\text{,ϵ}}\text{w}\text{ (}\text{Equation S}\text{5}\text{)}$$

## S1.2 Sign convention and prospective Clause Zero calibration

Eigenvectors are sign-indeterminate. Figure 3 loading profiles orient the γ = 1 cPCA reference to a positive source RSA loading (displayed as RSurfA in durum) and align gcPCA toward that reference. The frozen single-species response analyses instead use the same source-anchor-positive gcPCA orientation fixed without held-out responses. Archived global cPCA displays retain their original sign convention. None of these display conventions calibrates RAD against water potential; the prospective physical convention is defined separately below.

For prospective calibration, define drought intensity as I_drought = −Ψsoil and retain the registered trait-loading sign convention from the training fit. State the anticipated association direction under that convention before evaluation. Any alternative orientation based on physiology must be learned in training or independent calibration data and frozen before held-out projection. Distinguish data used to choose the convention from independent evaluation of the frozen coordinate. Report the signed association under that fixed convention, including a reversed or unresolved direction. A positive correlation produced by choosing the axis sign is not evidence for monotonicity.

Before the evaluation experiment, specify the target species and genotypes, developmental stage, substrate, drying trajectory, sampling schedule, instrument calibration and matched water-potential, phenotype and transcriptome measurements. Fix the physiological window in main-text Equations 3 and 4 from independent physiological evidence or calibration data, including rules for boundary uncertainty and failed measurements, without inspecting RAD associations or predictive results. Apply recorded measurement-quality and exclusion rules while blinded to these outcomes, and report every exclusion and protocol deviation. Design admissibility concerns these experimental and measurement conditions; it never depends on the observed RAD association with water status.

Plan several actually measured water-status levels or a sufficiently resolved drying trajectory within each genotype and block, with overlapping water-status ranges where genotype comparisons are intended. Include time-matched controls to distinguish water-status responses from developmental change. Replicate independent experimental units across the trajectory and justify their number from the planned precision and expected variability; no universal sample size or power is asserted here. State whether the unit is a plant, pot or plot, and retain the hierarchy of genotype, block, unit and repeated measurements. Repeated sensor readings and subsamples do not increase independent n. Destructive phenotyping requires separately allocated independent units at each sampling time.

Two nominal water treatments can yield many measured water potentials across units or time. Such measurements may inform calibration if their range and replication resolve the response within genotypes and blocks. In contrast, two treatment means, including two means per genotype, or only two distinct measured water-potential values do not establish the shape or monotonicity of a dose response.

Pre-specify one primary calibration analysis that estimates the RAD association with water status within genotypes and blocks and accounts for the independent unit and repeated measures. For adequately sampled strata, report signed Spearman correlations with cluster-aware uncertainty; a pooled Spearman correlation is descriptive only. When the design requires partial pooling, specify a hierarchical dose-response model with genotype and block effects, within-group water-status effects and unit-level dependence before analysis. Fix the model form, handling of sampling time and developmental stage, uncertainty procedure and sensitivity specifications before examining associations. State whether intervals condition on a frozen coordinate or propagate training-fit uncertainty. Report group-specific estimates, heterogeneity and departures from monotonicity. A claim of monotonic calibration must be supported by within-genotype and within-block responses, not by differences between pooled groups. Monotonicity imposed during fitting cannot serve as evidence that the observations are monotonic.

Report all planned units and strata, achieved water-status ranges, association direction and strength, uncertainty intervals, heterogeneity, missingness, exclusions and departures from the registered trajectory or window. Pre-specified sensitivity analyses assess alternative defensible calibration specifications or physiological-boundary uncertainty on the same reported cohort; they do not search for a favorable correlation cutoff or revise eligibility. Weak, absent, reversed or imprecise calibration remains a result. For a defined geometric target, Clauses A and B retain their registered algorithm and evaluation plan, while conclusions about drought plasticity are limited by that calibration. Neither public dataset permits this calibration or an empirical correlation-threshold sensitivity analysis.

## S1.3 Interpretation of the leading generalized eigenvalue

If $\text{λ}_{\text{max}}\text{≤1}$, no direction has foreground variance exceeding the ridge-regularized background variance under the specified quotient. This indicates no foreground variance enrichment under that extraction rule; it does not imply that a geometric direction is undefined.

The eigenvalue is reported as a descriptive geometric diagnostic, not an automatic stopping rule. Clauses A and B may evaluate a defined target under the pre-specified analysis plan; physiological interpretation remains subject to Clause Zero calibration.

## S1.4 Source-precision A. tauschii geometry and response diagnostics

The complete A. tauschii cohort comprised accessions 129–148, two treatments and two recorded replications, giving 80 records (Pour-Aboughadareh and Poczai, 2021b). The source experiment describes a randomized factorial design with two blocks. Treatment codes 1 and 2 were mapped to well-watered control and drought using the audited study mapping; the worksheet does not contain a treatment codebook. Different treatment pots were matched within accession and recorded replication, not treated as repeated measurements of one plant. All stored SRL, RDW, RSA, RV and RD values were retained without additional rounding. TRLcalc = SRL × RDW was recalculated for every record. For accession 131 in the first control replication, stored RDW = 0.01 gives TRLcalc = 101.6792, whereas the former extra rounding produced zero. Source precision does not resolve this product's physical units or instrument calibration. RD denotes the source-native field.

Both treatments used one pooled training mean and sample SD vector before separate within-treatment covariance estimation. gcPCA solved A v = λ(C + rI)v with r = 10⁻⁶. Eigenpairs were ordered by decreasing λ, vectors had unit Euclidean length and the first direction was oriented to nonnegative RSA. Exact-zero RSA used TRLcalc, RV and RD in that order. A separate 10⁻⁶ near-zero anchor diagnostic did not change vector selection. The full spectrum, eigengaps, condition numbers and normalized equation residuals were retained; the residual tolerance of 10⁻¹⁰ was a numerical check, not a scientific stability criterion. Covariances retain between-accession structure.

The formal Figure 3A cPCA scan was completed deterministically with the original implementation on the same source-precision inputs. A leading additive eigenvalue above 10⁻¹⁰ defined a feasible cPCA direction. All 40 entries in the archived formal gamma registry were retained. Figure 3C uses all 39 entries already frozen for durum; it lacks γ = 0.975 and was not refitted to equalize the grids. Near-duplicate 0.7 entries are retained registry entries, not independent replications. Figure 3A/C use absolute cosine. In B/D, cPCA at γ = 1 is oriented to positive RSA and gcPCA is aligned toward it. The native RSA-positive A. tauschii gcPCA vector is (0.184879, 0.563656, −0.773167, 0.224331); its Figure 3B display has the opposite sign.

The first direction of the implemented ratio formulation is not outside the cPCA family. With isotropic ridge, A v = λ(C + rI)v implies (A − λC)v = λr v. For the largest generalized eigenvalue, the same vector is a first cPCA direction at γ = λ. The corresponding weights here, 1.898843 for A. tauschii and 39.938692 for durum, lie outside the displayed 0.05–1 range. Figure 3 therefore compares the frozen settings rather than optimized cPCA performance. This relation is specific to this variance-ratio formulation and isotropic ridge (de Oliveira et al., 2025).

For response and cross-fit angular diagnostics, the frozen common reference was the old full-cohort pooled SD vector s₀ = (35.334076131407862, 58.800337928504533, 1.0901740367389725, 0.072522366693910878). For a training vector v and SD vector s, a = diag(s₀/s)v, k = ||a||₂ and q = a/k. Angles were acos(|q′q₀|), relative to the same preprocessing chain's full-cohort direction. The source-precision q was (0.187384, 0.557382, −0.764737, 0.263418), distinct from the native plotting vector. The leading two-dimensional common-metric spans were orthonormalized by QR and compared by SVD principal angles. A correspondence diagnostic recorded when the second vector was closer to the baseline first direction; it neither relabelled axes nor established eigenvalue crossing.

For each accession and replication, δnative = v′[(xD − xC)/s], δcommon = δnative/k and δCSD = δnative/SDC,train. The denominator SDC,train = sqrt(v′Cv) uses the unregularized training-control covariance. In the full source-precision fit, k = 1.0112924202 and SDC,train = 0.0899774057. The 40 matched contrasts were analysed with δnative ~ accession + replication, both as fixed effects with sum contrasts. The conditional mean interval was mean(δ) ± t0.975,19 sqrt(MSE/40); interaction and remaining error cannot be separated with one contrast per cell. The untruncated component (MSaccession − MSE)/2 was retained as a diagnostic, including negative values, without interpreting it as a literal negative variance or estimating heritability from two replications. No additional inferential p values were introduced.

The 2,000 accession-cluster bootstrap samples used seed 20260905 and R 4.3.3 with Mersenne-Twister, Inversion and Rejection. Each sampled accession copy retained both treatments and both replications under a distinct copy key. The fixed-axis bootstrap resampled full-cohort accession mean responses. Complete refitting repeated training scaling, covariances, all four eigenvectors and the sign rule, then evaluated responses in the resampled cohort. Percentile endpoints used R quantile type 7. The old and source-precision analyses used identical draw IDs; their paired differences are sensitivity summaries. These resampled responses are not out-of-sample validation. No draws failed or were replaced.

Each of 20 leave-one-accession-out folds trained on 76 records and projected the four held-out records with training-only centers, SDs, axes, signs and control-projection SDs. Replication-specific fits used 20 records per treatment and projected onto the other replication. Overlapping folds were not assigned independent-fold confidence intervals or p values. Ridge sensitivity retained η = 0, 10⁻¹⁰, 10⁻⁸, 10⁻⁶, 10⁻⁴, 10⁻³, 10⁻², 10⁻¹ and 1, where η = r/[tr(C)/4], plus the absolute baseline r = 10⁻⁶. Each preprocessing chain used its own C to convert η to r. None of these results was used to tune the ridge.

The fixed-axis common-metric interval was [0.101873, 0.185759], but the complete-refit interval was [−0.172771, 0.196331]. Across-replication response Pearson correlation was −0.306708; 13/20 accessions had concordant signs, and the two cross-replication projections had opposite signs. The bootstrap first-direction angle median was 6.3461° and its 97.5th percentile was 85.8244°. Omitting accession 131 rotated the direction by 74.9249°. Thus the corrected analysis retains evidence of a conditional mean response without establishing stable accession-specific responses or a stable first direction (Table S7). These conclusions are conditional on the selected species, operational traits and two observed replications. The stored bootstrap, exclusion and ridge analyses were not rerun for this figure integration.

For Figure S1B, native local coefficients were mapped as diag(sglobal/slocal)v into the frozen global pooled-SD coordinate system, then restricted to RSA, RV and RD and renormalized. The common columns and SDs were checked against the archived 720-record inputs. The global PCA was not refitted. Table S1 updates only its two A. tauschii rows; changes combine source-precision adoption and correction of the previous local/global coefficient-coordinate mismatch. The five full-data rows, archived global score distributions and full-data cPCA sweep retain their original descriptive preprocessing. The separate 12-species accession-by-treatment mean QC already used source precision and was neither rerun nor reranked.

The generalized eigenvalue is a regularized variance ratio, not a mean treatment effect, a loading-stability statistic or a performance score directly comparable with an additive cPCA eigenvalue. Neither the source-precision correction nor a positive eigenvalue establishes physiological calibration.

The wheat dataset contains 720 records nested in 180 accessions, each with two records per treatment. The 360/360 counts in Figure S1A describe observations. Single-species response analyses preserve the accession-by-replication matching of different treatment pots. The separate durum experiment uses independent, unpaired pots within genotype-by-treatment cells. These sampling structures are retained in their respective uncertainty procedures.

## S1.5 Species-level cPCA quality-control screen

To replace the earlier restricted convenience subset, we evaluated all 12 source-defined species using their complete accession ranges. For each species, the two recorded replicates were averaged within each accession-by-treatment combination, and pooled common standardization was re-estimated within that species cohort. cPCA was fitted to the leading eigenvector of Sigma-D minus gamma Sigma-C over a frozen grid from gamma = 0.05 to 1.00 in increments of 0.025. A cPCA direction was defined as feasible when the leading contrast eigenvalue exceeded 1 x 10^-10. Axis sign was fixed by making the first loading with maximum absolute magnitude positive.

Sampling stability was evaluated using 2,000 accession-cluster bootstrap resamples per species. Each sampled accession carried both treatment means, and preprocessing and cPCA fitting were repeated within the resample. Eligibility for the single-species illustration required at least 15 accessions, a positive gamma = 1 leading contrast in the accession-mean fit, and positive gamma = 1 leading contrasts in both replication-specific fits. Eligible species were ranked, in order, by the number of feasible gamma settings, the absolute loading cosine between gamma = 0.70 and 1.00, the 2.5th percentile of the gamma = 1 bootstrap loading cosine, the replication-1 versus replication-2 loading cosine, the gamma = 1 bootstrap positive fraction, accession count, and species name. All 12 species were retained in the reported screen. The screen selected a cohort for a descriptive geometric illustration; it was not used as an inferential test of RAD or the downstream transcriptomic framework.

## S1.6 External durum wheat reference

We used the public whole-root measurements associated with Urbanavičiūtė et al. (2022a), archived at Zenodo (Urbanavičiūtė et al., 2022b; doi:10.5281/zenodo.5883299; CC BY 4.0). The panel comprised six genotypes, two water treatments and three independent pots per genotype-by-treatment cell. All 36 pots were retained. Total root length, root surface area, root volume and mean diameter were measured directly, in cm, cm², cm³ and mm, respectively. For display, source RSA is RSurfA and the directly measured mean-diameter field RD is RDmean; the original fields remain unchanged. Different treatment pots were not paired, and replicate labels were not treated as confirmed blocks. Published Table 2 values were checked separately for length, area and volume; its RDW field denotes root dry weight, not diameter.

The numerical specification was frozen before these revision-stage fits; it was not an original study preregistration. Stored trait precision was retained without additional rounding or logarithmic transformation. Within each training set, both treatments were standardized using their pooled means and sample standard deviations. Separate treatment covariance matrices were then estimated. They retained between-genotype structure because genotype means were not removed. gcPCA used the leading solution of ΣD v = λ(ΣC + rI)v, with fixed r = 10⁻⁶. cPCA used ΣD − γΣC with primary γ = 0.7. The original gamma registry and the additional γ = 1 diagnostic were retained. Each direction had unit Euclidean length and positive RSurfA loading (source RSA). Exact-zero anchors would fall back to TRL, RV and RDmean (source RD), in that order. No direction was oriented using held-out responses or the full-data direction.

Pot resampling drew three observations with replacement within each of the 12 genotype-by-treatment cells. All 2,000 draws retained the six-genotype composition, with seed 20260906. The same draws were used for both extraction methods. Complete refitting repeated scaling, covariance estimation and axis estimation. Fixed-axis intervals held the full-data preprocessing and direction unchanged. Intervals are pointwise 2.5th and 97.5th percentiles, using R quantile type 7, and are conditional on the observed genotypes. Treatment effects are drought-minus-control means within each genotype, followed by an equal-genotype average. No ordinary paired-pot test or interval from overlapping validation folds was used.

Leave-one-genotype fits used 30 training pots and projected the six held-out pots without refitting. The primary held-out response was expressed in training-control projection SD units, with every fitted quantity estimated in training. For descriptive cross-fit comparison, let s₀ denote full-panel pooled SDs and s denote training SDs. We used a = diag(s₀/s)v, k = ||a|| and q = a/k. Common-metric scores equal native training-standardized scores divided by k; directional angles compare common-metric unit vectors. The full-panel reference only defines this post hoc metric. It does not determine training, sign, or the primary held-out response. The six overlapping training folds and 36 leave-one-pot fits are influence diagnostics, not independent validation experiments. Archived held-out perturbation checks document unchanged training transformations and fitted directions when held-out traits change, in both public-data branches (deterministic_tests.csv).

Ridge sensitivity retained the entire frozen grid η = r/[tr(ΣC)/4] from 0 to 1, plus the absolute baseline ridge. All grid values are reported in Table S6d. At η = 1, the ridge equals the mean background diagonal variance, constituting a strong stress test. This endpoint remains part of the composite judgement. The frozen directional-angle limits and all other criteria are reproduced in Table S6e without modification.

## Supplementary results

Root surface area and volume decreased in all six genotypes. Their equal-genotype contrasts were −159.818 cm² and −5.440 cm³, with pointwise 95% intervals of [−181.524, −138.620] and [−5.949, −4.926], respectively. Length and diameter contrasts are also reported (Table S6a); the length response was positive in Mv PELSODUR. Means matched the published Table 2 precision in 33/36 comparisons and SDs in 36/36. Three mean discrepancies slightly exceeded half a unit of the published final digit. All source values were retained; intermediate rounding or transcription cannot be established as the cause (Table S6g).

The gcPCA leading eigenvalue was 39.939, with relative gap 0.986 and background condition number 1096.345 (Table S6b). All 2,000 bootstrap fits were solved without recorded numerical degeneracy. The mean contrast was 0.239 in the common metric and 7.467 training-control SD units. The native projected control SD was 0.032013, so the latter value must not be interpreted independently of its denominator. The fixed-axis common-metric interval excluded zero, whereas the complete-refit interval was [−0.155, 0.312]. All six held-out genotype responses were positive. Maximum genotype and pot exclusion angles were 1.107° and 0.830°, respectively. However, the bootstrap angle's 97.5th percentile was 41.331°, exceeding the frozen 30° criterion. At η = 1, the angle reached 59.247° and the mean contrast changed sign. cPCA at γ = 0.7 also had a complete-refit interval spanning zero and only four agreeing genotype holdouts. Neither method met its complete frozen stability criteria. Individual exclusions assess local influence; complete resampling changes the relative representation of several pots while re-estimating the coordinate. The favorable exclusion results and the broader bootstrap uncertainty therefore describe complementary aspects of robustness. Table S6e records the full criteria, including sensitivity to strong regularization.

## **S2. Relative topology deviation and reference module projection**

Relative topology deviation is estimated from condition-specific TOMs on matched module gene sets. The implemented score is the Frobenius difference divided by the reference Frobenius norm, with the numerical denominator safeguard made explicit below. This definition retains the algorithm used for the controlled numerical results.

$$\text{Δ}{\tilde{\text{R}}}_{\text{k}}\text{=}\frac{\left\| \text{T}_{\text{fg}}^{\left( \text{k} \right)}\text{−}\text{T}_{\text{bg}}^{\left( \text{k} \right)} \right\|_{\text{F}}}{\max\left( {10}^{-6}, \left\| \text{T}_{\text{bg}}^{\left( \text{k} \right)} \right\|_{\text{F}} \right)}\text{ }\text{ }\text{ (}\text{Equation S}\text{6}\text{)}$$

Here, $\text{T}_{\text{fg}}^{\left( \text{k} \right)}$ and $\text{T}_{\text{bg}}^{\left( \text{k} \right)}$ are module submatrices extracted from condition-specific TOMs estimated on the same gene universe. The Frobenius norm includes both symmetric off-diagonal entries and the diagonal. With WGCNA unit diagonals, diagonal differences are zero in the numerator, while the diagonal contributes the module size m to the squared reference norm in the denominator. Thus Equation S6 uses full-matrix normalization; the off-diagonal calculation in Equation S7 below is a conceptual size argument, not the score used for the figures.

## S2.1 Module size and normalization

A raw Frobenius distance scales with the number of TOM entries. For a conceptual calculation, suppose a module has size $\text{m}$ and foreground-background off-diagonal differences are zero-mean fluctuations with common variance $\text{σ}^{\text{2}}$. The expected squared off-diagonal distance then scales with the number of off-diagonal entries.

$$\text{E}\left[ \left\| \text{T}_{\text{fg}}\text{−}\text{T}_{\text{bg}} \right\|_{\text{F}\text{,off}}^{\text{2}} \right]\text{≈}\text{m}\left( \text{m}\text{−1} \right)\text{σ}^{\text{2}}\text{ }\text{ }\text{ (}\text{Equation S}\text{7}\text{)}$$

The expected raw Frobenius distance increases with module cardinality even when no systematic topology change is present. Because weighted gene co-expression network analysis (WGCNA) modules can vary substantially in size, an unstandardized distance would make larger modules appear more perturbed simply because they contain more entries.

Module size also depends on module-cutting and merging choices. Relative normalization puts the difference on the scale of the same module’s reference topology, but this rescaling alone cannot establish comparable sampling behavior across module sizes or network configurations.

## S2.2 Interpretation and estimation conditions

The ratio is dimensionless but is not module-size invariant. Baseline connectivity and the diagonal contribution affect its scale, especially for small or weakly connected modules. For the retained unit-diagonal TOMs, the reference norm is at least √m, so the 10⁻⁶ floor is inactive. A zero-diagonal or other near-zero-baseline variant would be a different specification requiring its own predeclared safeguard and sensitivity assessment; the numerical floor alone would not make such a ratio informative.

Sampling noise in estimated correlations and TOMs contributes to $\text{Δ}{\tilde{\text{R}}}_{\text{k}}$. Interpretation must account for independent sample counts in each condition, unequal sample sizes, module size, baseline connectivity, WGCNA power, signed or unsigned network construction, and the TOM type and denominator convention. Use identical gene sets and gene order across conditions and record the network-wide gene universe, because shared-neighbor TOM values can depend on genes outside a module.

ΔR is a relative topology-deviation summary, not a module-preservation test or evidence of a specific mechanism. The controlled scenarios illustrate the score and its penalty mapping. Bootstrap or permutation calibration of ΔR and comparisons with established module-preservation diagnostics have not been performed for these scenarios; they are specified as future empirical calibration in S2.5.

## S2.3 Current illustrative implementation

The archived controlled scenario uses 150 samples per condition and 770 genes in ten predefined contiguous gene blocks, with the sizes listed in Figure 4. Modules are assigned by construction rather than detected from the data. The generator centers and scales genes separately in the reference and foreground matrices. Using R 4.3.3 and WGCNA 1.73, the implementation estimates a TOM on all 770 genes separately for each condition, then extracts the predefined module submatrices. The calls specify power=6 and networkType="unsigned" and inherit corType="pearson", TOMType="signed" and TOMDenom="min". The TOM-type option and network-type option are distinct settings.

The retained code calls moduleEigengenes on foreground expression alone, using the predefined module labels. Its default SVD-based first-component calculation scales genes and aligns the score along the foreground module-average expression; the returned scores are then centered and scaled across foreground samples. This path does not estimate a reference eigengene or reference loadings for projection. Reference and foreground TOM estimation is separate from eigengene estimation.

The archived wrapper also renames returned eigengene columns M1 through M10 by position. WGCNA orders the supplied character labels as M1, M10, M2, …, M9, so the eigengene column renamed M2 represents gene module M10, and renamed columns M3 through M10 represent gene modules M2 through M9. Topology deviations retain numerical gene-module order. This positional relabeling does not preserve module identity in the eigengene-to-topology join. The screening scenario additionally constructs its M3×M4 predictor as a noisy copy of M1×M2 and defines the response from its labeled predictors. The retained screening labels and results therefore describe this configured illustration, not validation of correctly matched module-specific weighting or reference projection. Figures 4 and 5 retain the actual gene-module topology values and assigned index-based weights.

## S2.4 Prospective reference projection

At every model-training level, define the reference modules using only the designated well-watered reference training samples, or use quantities frozen from independent calibration with their origin recorded. Fix gene identifiers and order, the outcome-blind gene QC and preprocessing rules, network and TOM settings, module-cutting and merging rules, and membership. Learn all required gene transformation parameters, reference gene means and positive sample standard deviations, PC1 loadings and orientation from that reference training data. Match eigengene columns and topology weights by module identifiers rather than by returned column position.

For each module, apply the stored gene preprocessing, subtract the reference mean for each aligned gene, divide by its stored reference sample standard deviation, multiply by the stored unit-norm PC1 loading vector and apply the fixed sign. Choose that sign against the reference module-average expression, with a predeclared deterministic loading anchor for ties. Apply this frozen transform to drought, validation and test samples without fitting a foreground PC1 or using held-out outcomes to orient it. This gene-level transformation precedes the separate centering and scaling of projected eigengenes within each model-training partition in S7.1; those eigengene means and scales are also frozen for validation and test samples.

Separately estimate reference and drought TOMs from their respective observations within the current training partition, on the same training-defined gene universe and with the same network/TOM configuration. Extract matching reference-module gene sets in the same order and compute Equation S6. Freeze the resulting topology weights for validation and test prediction; a TOM is not projected by the eigengene loading vector. Holding these quantities fixed across Clause A pseudo-responses requires the recorded outcome-blind, external or independent-calibration basis specified in S6; outcome-dependent versions must be repeated.

## S2.5 Calibration for future empirical applications

Before empirical application, specify the resampling design and count, reference module sets, gene universe, primary network/TOM configuration and reported uncertainty summaries. Bootstrap independent biological units within the treatment and block structure, carrying repeated observations or matched treatment measurements together when the design requires it. Re-estimate condition-specific TOMs on the same gene sets and report uncertainty in ΔR and the resulting weights, together with module sizes and independent sample counts. Treatment-label permutation may supply a no-condition-difference reference only when the design justifies exchangeability; it is not automatically valid for observational or blocked data.

Keep the primary network configuration fixed during calibration and register any sample-size balancing, power, signed/unsigned-network or TOM-variant sensitivity comparisons in advance. Compare ΔR with WGCNA modulePreservation diagnostics, including Zsummary and medianRank, on the same reference module gene sets (Langfelder et al., 2011). Interpret Zsummary alongside module size and medianRank as a relative ranking, without importing a preservation cutoff into ΔR. A predeclared equal-weight fit, with all pairwise penalty multipliers set to one, will assess sensitivity to the proposed weighting using the same training partitions, module basis and evaluation units. These are future calibration and sensitivity analyses, not results of the present controlled illustrations.

## S2.6 Coverage of the reference basis

A fixed reference basis makes coordinates comparable and keeps the predictor definition independent of drought-outcome screening. Topological remodeling of a represented reference module changes its continuous penalty; it does not automatically delete the module. In contrast, drought-emergent modules and recombinations not represented by that basis can be missed, so predictive conclusions are restricted to the predefined basis. Projection and ΔR do not establish conserved biological identity. De novo, consensus or additional-module exploration is a separate future extension requiring its own outcome-blind training definition and validation; omitted modules must not be added retrospectively to an already evaluated model.

## **S3. Pre-specified continuity weighting of candidate module pairs**

The framework converts module-level relative topology deviation into a proposed continuous topology-informed penalty prior for each candidate pairwise product. For modules $\text{i}$ and $\text{j}$, the weight is defined as follows.

$$\text{Λ}_{\text{ij}}\text{=}\frac{\text{1}}{\text{1+Δ}{\tilde{\text{R}}}_{\text{i}}\text{+Δ}{\tilde{\text{R}}}_{\text{j}}}\text{ }\text{ }\text{ (}\text{Equation S}\text{8}\text{)}$$

The least absolute shrinkage and selection operator (LASSO) penalty multiplier for this candidate term is then defined as the inverse of the prior weight.

$$\text{p}_{\text{ij}}\text{=}\text{Λ}_{\text{ij}}^{\text{−1}}\text{=1+Δ}{\tilde{\text{R}}}_{\text{i}}\text{+Δ}{\tilde{\text{R}}}_{\text{j}}\text{ }\text{ }\text{ (}\text{Equation S}\text{9}\text{)}$$

This mapping is not the only possible route from topology to penalty. It is selected as a minimal pre-specified rule because it introduces no fitted rate constant: Λij is bounded on $\text{(0,1]}$, continuous, differentiable, monotone decreasing, and convex as a function of the non-negative summed deviation.

## S3.1 Why a minimal pre-specified mapping is used

Other monotonic functions could also be constructed. For example, exponential mappings of the following form require a decay constant unless the constant is fixed in advance.

$$\text{Λ}_{\text{ij}}\text{=exp}\left[ \text{−}\text{α}\left( \text{Δ}{\tilde{\text{R}}}_{\text{i}}\text{+Δ}{\tilde{\text{R}}}_{\text{j}} \right) \right]\text{ }\text{ }\text{ (}\text{Equation S1}\text{0}\text{)}$$

Hard-threshold mappings require a cutoff and create discontinuities near that cutoff. Power-law alternatives introduce additional shape choices unless those choices are fixed in advance. The inverse-linear mapping is used here because it is a minimal analytical rule that provides continuous, monotonic weighting without outcome-adaptive tuning.

## S3.2 Biological scope of the continuity weight

$\text{Λ}_{\text{ij}}$ expresses a preference for smaller relative topology deviations when assigning penalties. It is not a preservation probability, stability proof, cell-composition correction or automatic eligibility rule. Strong deviation can reflect estimation noise or biologically meaningful remodeling. Reference modules remain in the predefined basis subject to the shared outcome-blind availability rules; increasing ΔR changes their pairwise penalties continuously and creates no additional hard cutoff. Predictive benefit over equal weighting remains to be assessed in the future sensitivity analysis in S2.5.

## **S4. Fixed-regularization resampling for candidate stability**

The framework summarizes feature-level retention under repeated subsampling at a fixed LASSO regularization level. Locking $\text{λ}$ separates subsampling-driven stability from variability introduced by re-tuning the regularization parameter within every resample.

Let ${\hat{\text{β}}}^{\left( \text{s} \right)}\left( \text{λ} \right)$ denote the LASSO coefficient vector estimated on subsample $\text{s}$. If $\text{λ}$ is re-selected by cross-validation in every subsample, the inclusion probability of feature $\text{k}$ reflects two sources of variation: variation caused by subsampling and variation caused by the locally selected $\text{λ}^{\left( \text{s} \right)}$.

$$\text{Ω}_{\text{k}}^{\text{ReCV}}\text{=}\frac{\text{1}}{\text{S}}\sum_{\text{s}\text{=1}}^{\text{S}} \text{I}\left[ {\hat{\text{β}}}_{\text{k}}^{\left( \text{s} \right)}\left( \text{λ}^{\left( \text{s} \right)} \right)\text{≠0} \right]\text{ }\text{ }\text{ (}\text{Equation S}\text{1}\text{1}\text{)}$$

This can inflate or deflate feature retention independently of the structural stability of the predictor-response relationship. In the controlled scenario, one $\lambda_{1se}$ is selected on the original-predictor matrix and kept fixed across subsamples. Figure 6A,B show this original-predictor regularization stage; Figure 6C,D use original-feature retention from the augmented target-plus-matched-decoy fits in S5, with duplicated penalty factors and the same subsampling schedule. In any empirical held-out performance assessment, 'global' must mean within each outer training partition; module construction, weights, standardization, and λ selection must not use the held-out partition. For prospective Clause A, λ selection is repeated for each pseudo-response before locking within that response's stability subsamples (S6).

$$\text{Ω}_{\text{k}}\text{=}\frac{\text{1}}{\text{S}}\sum_{\text{s}\text{=1}}^{\text{S}} \text{I}\left[ {\hat{\text{β}}}_{\text{k}}^{\left( \text{s} \right)}\left( \text{λ}_{\text{1se}} \right)\text{≠0} \right]\text{ }\text{ }\text{ (}\text{Equation S1}\text{2}\text{)}$$

Using $\text{λ}_{\text{1se}}$ rather than $\text{λ}_{\text{min}}$ favors a sparser model and reduces retention of weak collinear terms that arise from marginal fit gains. This is consistent with the framework's aim of describing stable candidate module pairs under conservative, pre-specified conditions.

## S4.1 Penalized fitting objective

For each subsample $\text{s}$, the model is fitted by minimizing the following objective under locked $\text{λ}_{\text{1se}}$ and fixed penalty multipliers $\text{p}_{\text{k}}$.

$${\hat{\text{β}}}^{\left( \text{s} \right)}\text{=arg}\min_{\text{β}_{\text{0}}\text{,}\text{β}}\left\{ \frac{\text{1}}{\text{2}\text{n}_{\text{s}}}\left\| \text{Y}_{\text{RAD}}^{\left( \text{s} \right)}\text{−}\text{β}_{\text{0}}\text{1}\text{−}\text{X}^{\left( \text{s} \right)}\text{β} \right\|_{\text{2}}^{\text{2}}\text{+}\text{λ}_{\text{1se}}\sum_{\text{k}} \text{p}_{\text{k}}\left| \text{β}_{\text{k}} \right| \right\}\text{ }\text{ }\text{ (}\text{Equation S1}\text{3}\text{)}$$

Here, $\text{n}_{\text{s}}$ is the number of samples in subsample $\text{s}$, and $\text{p}_{\text{k}}$ is the fixed topology-derived penalty multiplier for predictor $\text{k}$.

## S4.2 Algorithm for the controlled illustration

1. Perform 10-fold cross-validation on the full predictor matrix $\left( \text{X}\text{,}\text{Y}_{\text{RAD}} \right)$ using the pre-specified topology penalty factors.
2. Extract $\text{λ}_{\text{1se}}$ and keep it fixed for all subsequent subsampling iterations.
3. For each iteration $\text{s}\text{=1,…,}\text{S}$, draw a 50% subsample without replacement.
4. Fit the penalized LASSO model using locked $\text{λ}_{\text{1se}}$ and fixed penalty factors.
5. Record whether each feature has a non-zero coefficient.
6. Estimate $\text{Ω}_{\text{k}}$ as the fraction of subsampling iterations in which feature $\text{k}$ is retained.

## S4.3 What the retention frequency does and does not show

$\text{Ω}_{\text{k}}$ is an operational retention measure under fixed analytical conditions. The Ω≥0.60 reference is an illustrative triage setting, not a calibrated significance or error-control cutoff. It is not a discovery claim and does not establish biological coordination. It feeds target-decoy candidate screening and Clause A, but does not select U_B or gate Clause B. Equations S11–S13 describe the retained controlled screening implementation; they do not demonstrate the hierarchical term inclusion required for the separate prospective Clause B fit in S7.

## **S5. Controlled target-decoy scenario of the pairwise module space**

The framework contains individual module eigengenes and their second-order Hadamard products. Because these terms can be highly collinear, feature retention alone cannot distinguish a stable candidate from a correlated alternative. The controlled target-decoy scenario therefore asks whether an original term is retained more often than a matched decoy under the same declared penalty conditions. This is a controlled screening illustration, not a formal false discovery rate (FDR) guarantee.

## S5.1 Candidate pairwise module space

Let $\text{M}$ denote the matrix of module eigengenes. For prospective use, each eigengene is centered and divided by its training sample standard deviation before constructing unordered pairwise products; training parameters are frozen for validation and test samples. Equation S14 specifies the same first-order plus pairwise basis as main-text Equation 6. The frozen controlled screening outputs are not recomputed under this prospective specification.

$$\text{X}\text{=}\left[ \text{M}_{\text{1}}\text{,…,}\text{M}_{\text{K}}\text{,}\text{M}_{\text{1}}\text{∘}\text{M}_{\text{2}}\text{,…,}\text{M}_{\text{i}}\text{∘}\text{M}_{\text{j}}\text{; 1≤i<j≤K} \right]\text{ }\text{ }\text{ (}\text{Equation S1}\text{4}\text{)}$$

Here, $\text{∘}$ denotes element-wise multiplication and only unordered pairs 1≤i<j≤K are included. Self-products and duplicate symmetric terms are excluded, giving K+K(K−1)/2 predictors before decoy augmentation; for K=10, this is 55 predictors. Individual module eigengenes represent module-state variables, whereas Hadamard products represent candidate pairwise coordination terms. These products are statistical predictors and should not be interpreted as physical molecular interactions. The separate 2,500-term Figure 8 triage draw is a high-dimensional controlled input and is not the K=10 topology scenario count.

## S5.2 Matched decoys in the controlled scenario

The controlled code constructs matched competitors from centered X by retaining its singular values and right singular vectors and replacing the left singular directions with an orthonormal basis obtained from random Gaussian vectors projected away from the original column space. No RAD response is used in this construction. The covariance comparison is descriptive and does not establish the swap exchangeability required for Model-X inference.

This step is not intended to prove exact Gaussianity, Model-X validity, or exchangeability. The matched decoy supplies a structured competitor for each original feature under the same regularization and topology conditions in the controlled scenario.

## S5.3 Retention frequencies for original and matched-decoy terms

The original and decoy predictors are concatenated as follows.

$$\text{X}_{\text{aug}}\text{=}\left[ \text{X}\text{,}\tilde{\text{X}} \right]\text{ }\text{ }\text{ (}\text{Equation S1}\text{5}\text{)}$$

The locked-$\text{λ}$ subsampling protocol from Supplementary Methods S4 is applied to $\text{X}_{\text{aug}}$. Each original feature $\text{X}_{\text{k}}$ receives retention frequency $\text{Ω}_{\text{k}}$, and its matched decoy ${\tilde{\text{X}}}_{\text{k}}$ receives ${\tilde{\text{Ω}}}_{\text{k}}$. The controlled target-decoy statistic is then defined as follows.

$$\text{W}_{\text{k}}\text{=}\text{Ω}_{\text{k}}\text{−}{\tilde{\text{Ω}}}_{\text{k}}\text{ }\text{ }\text{ (}\text{Equation S1}\text{6}\text{)}$$

A positive $\text{W}_{\text{k}}$ indicates that the original term is retained more often than its matched decoy under the same analytical constraints. A value near zero indicates no clear advantage over the matched competitor under this controlled scenario.

## S5.4 Nominal candidate boundary for the controlled scenario

For the controlled Figure 7 screen, Equation S17 is evaluated over the finite set 𝒯 of distinct observed absolute W values at least 0.60 among pairwise terms. The reverse-to-forward count ratio defines a nominal triage boundary, not a knockoff discovery threshold. If this search returns no value, the archived display uses the larger of the maximum pairwise W and 0.60 as a fallback; a prospective screen must declare its empty-set rule in advance.

$$\text{τ}\text{=min}\left\{ \text{t}\text{∈𝒯:}\frac{\left| \text{\{}\text{k}\text{:}\text{W}_{\text{k}}\text{≤−}\text{t}\text{\}} \right|}{\text{max}\left( \text{1,}\left| \text{\{}\text{k}\text{:}\text{W}_{\text{k}}\text{≥}\text{t}\text{\}} \right| \right)}\text{≤}\text{q} \right\}\text{ }\text{ }\text{ (}\text{Equation S1}\text{7}\text{)}$$

Here q=0.05 is a nominal count-ratio setting, not an FDR level. The separate 2,500-term Figure 8 illustration uses directly generated retention-like scores and fixed τ=0.550, not estimated inclusion frequencies from S4 or the output of Equation S17. Terms meeting the nominal boundary are nominated for Clause A only and do not restrict Clause B inputs.

## S5.5 Interpretive scope and requirements for future empirical implementation

No finite-sample FDR, Knockoff+, fixed-X or Model-X guarantee is claimed. The fixed-seed displays do not estimate repeated-study type-I error, power, interval coverage or selection stability of the full procedure. End-to-end validation remains future work across sample size, module correlation, predictor-to-sample ratio, signal strength, topology disruption, cell-composition confounding and measurement error. The independent-test-set design analysis in S7.5 answers a separate conditional Clause B question and does not validate Clause A or target-decoy screening.

## S5.6 Interpretation boundary

Target-decoy screening does not establish a causal biological interaction. It nominates candidates for Clause A under the declared construction, penalty and resampling scheme. Clause B separately evaluates the specified predictor basis over U_B, regardless of τ, Ω or Clause A survival. Measurement-sensitivity analysis and biological validation concern later interpretation, not retrospective selection of the Clause B module universe.

## **S6. Clause A: testing candidate persistence against a structured null**

The prospective Clause A reference is the global null of no RAD association with the module predictor space after adjustment for pre-specified nuisance effects. Define $U_{A}$ before RAD inspection as all eligible unordered module pairs from the outcome-blind training reference. The augmented fit may include base terms, but the candidate comparison family is $U_{A}$; the 39 terms in Figure 8 do not define it.

Within each outer training partition, specify a non-saturated nuisance design $Z$ for relevant genotype or accession, block, batch or sampling-time effects. Fit the nuisance-only model to the observed RAD target. Equation S18 adds its fitted values to the permissibly permuted residuals; the superscript + denotes the Moore–Penrose inverse, and the identity transformation gives the observed response.

$Y_{RAD}^{(b)}=H_{Z}Y_{RAD}+P_{b}\left( I-H_{Z} \right)Y_{RAD}, H_{Z}=ZZ^{+}$ (Equation S18)

Choose permissible transformations from the experimental design, not from observed associations. Repeated observations move together as whole biological units among exchangeable units with compatible observation schedules within the declared blocks; the same unit boundaries apply to training partitions, tuning folds and subsamples. The observed nuisance fit defines the add-back transformation in Equation S18. Each resulting response then enters the same pre-specified algorithm, which repeats every outcome-dependent preprocessing, tuning, screening, λ selection and applicable τ or other boundary choice. Nuisance terms are re-fitted without penalization in each subsequent fit. Predictor definitions, topology weights, decoy draws and random resampling schedules may remain fixed only when their construction is outcome-blind, externally fixed or independently calibrated, with that basis recorded. Outcome-dependent versions are repeated. All fitting stays within the relevant training data.

$\Omega_{k}^{(b)}=\frac{1}{S}\sum_{s=1}^{S} I\left( \beta_{k}^{(s,b)}\left( \lambda_{1se}^{(b)} \right)\neq0 \right)$ (Equation S19)

For response b, select $\lambda_{1se}^{(b)}$ by the same inner tuning rule and hold it fixed only across that response's stability subsamples. Let $C_{b}$ be the subset of $U_{A}$ obtained by repeating the complete candidate screen, including any data-derived τ. The observed response uses $b=0$. Compute the maximum over $C_{b}$; define the maximum as zero when $C_{b}$ is empty. An empty $C_{0}$ yields no Clause A candidates and does not affect Clause B.

$M_{null}^{(b)}=\max_{k\in C_{b}}\Omega_{k}^{(b)}$	(Equation S20)

The default prospective budget is B=1,000 uniformly sampled permissible permutations; enumerate all non-identity transformations instead if fewer are available and report the actual B. Sort the B maxima increasingly. To specify the discrete upper tail, use the order-statistic ceiling below rather than an interpolated percentile.

$\Omega_{\mathrm{null},95}=M_{\mathrm{null},(r)}, r=\left\lceil0.95\left( B+1 \right) \right\rceil$ (Equation S21)

For an observed candidate in $C_{0}$, the upper-tail permutation probability is one plus the number of null maxima at least as large as its observed retention, divided by B+1. Ties count in the upper tail. At nominal α=0.05, Equation S22 uses a strict exceedance; if r>B the ceiling is infinite. Report Monte Carlo uncertainty when permutations are sampled.

$\Omega_{k}^{(0)}>\Omega_{\mathrm{null},95}$	(Equation S22)

Validity depends on justified exchangeability and nuisance adjustment for the actual design. Freedman–Lane residual permutation is generally approximate with fitted nuisance effects and is not automatically exact under arbitrary dependence (Winkler et al., 2014). The maximum supplies a global-null reference for this full selection procedure, not proof of a unique joint effect, strong familywise error control under partial alternatives or FDR control. This prospective test has not been executed in the archived illustrative code.

## **S7. Clause B prospective assessment of the specified predictor basis**

Clause B assesses predictive adequacy of the specified first-order plus pairwise Hadamard predictor basis under the declared fitting and evaluation procedure, relative to a pre-specified higher-capacity comparator. It does not identify general interaction order. Figure 9 contains directly constructed surfaces, errors and attribution quantities, not nested model fitting, cross-validation performance or SHAP outputs. No empirical model-fitting protocol has been executed; S7.5 evaluates the declared frozen-model interval using simulated independent test sets.

Define U_B by pre-specified, outcome-blind transcriptome QC and reference-module rules, fitted in the corresponding training data, with base eigengene matrix $\text{M}$. The well-watered training samples define reference-module membership, network parameters, eigengene loadings and orientation, TOM settings and merging rules. Project that reference into drought and held-out samples. Both comparators receive the same full U_B and the same observational units. Clause A survivors, target-decoy τ, retention frequency Ω and RAD association screening do not define U_B. Any technical module-availability rule must be outcome-blind and applied equally to both comparators; ΔR remains continuous and creates no additional hard cutoff. An empty Clause A candidate set does not skip Clause B.

## S7.1 Training transformations and hierarchical term inclusion

Within every model-training partition, center each base eigengene and divide by its sample standard deviation, then construct M_i∘M_j only for i<j. Apply those frozen means, scales and reference projections to the corresponding validation or test samples. Exclude no module on the basis of target association. Zero-variance or missing projections follow the pre-specified outcome-blind availability rule shared by both models. Any product centering or scaling must also use training data only and be frozen; for this simple specification, products receive no additional rescaling after multiplication.

Fit an intercept, every admissible base main-effect term, and the unordered pairwise products. Keep the intercept and all base main-effect terms unpenalized; apply sparse L1 penalization only to products, with the pre-specified continuous topology multipliers from S3. This enforces hierarchical term inclusion because both parent terms remain in the model when a product is used. It does not require their estimated coefficients to be non-zero. Ordinary glmnet LASSO has no automatic strong-heredity guarantee. A zero penalty factor leaves a term unpenalized; software penalty-factor normalization and any internal standardization must be recorded consistently (Friedman et al., 2010). The S4 controlled LASSO values and Equations 8 and S13 remain descriptions of the original candidate-screening illustration, not results from this prospective fit.

This simple specification requires a full-rank, non-saturating unpenalized design block in every training partition, including inner tuning folds. The training sample count must exceed the rank of the intercept, base main effects and any pre-specified unpenalized nuisance terms. Rank deficiency, near-singularity, saturation or unstable predictions must be reported. The large product block can still cause non-unique sparse coefficients; solver convergence and deterministic tie handling must be declared, and selected terms are not uniquely identified biological effects. If these feasibility conditions fail, this Clause B specification is non-evaluable. Do not drop modules by outcome screening or silently introduce dimension reduction or another penalty; any alternative requires a new prospectively specified analysis.

## S7.2 Comparator and evaluation choices to lock before the experiment

The higher-capacity comparator is a pre-specified gradient-boosted regression-tree procedure on the same standardized base eigengenes U_B. Its actual capacity and tuning range must be chosen for the future experiment before outcome inspection. The Equation 6 fit and this comparator are not claimed to be nested function classes. Higher capacity may capture squares, nonlinear main effects, thresholds or localized response structure omitted by the specified basis; an advantage therefore does not identify general interaction order. The depth-2/depth-3 labels in Figure 9 are only schematic capacity labels, not this empirical comparison.

Before data collection or outcome access, lock the independent sampling unit and cluster hierarchy, target population, trait and transcriptome QC, reference-module construction and projection, primary RMSE and target scale, training and independent-test allocation, outer grouping and partitions, repetition counts, inner tuning splits, hyperparameter search ranges, selection and tie rules, random seeds, software versions and computation budget for both procedures. Use the same splits and primary loss for tuning; model-specific hyperparameter grids may differ but their search rules and budgets cannot change after evaluation. Specify missing-data and failed-fit handling, including how both models retain identical evaluation units. No test result may select a target direction, module set, model class, hyperparameter or margin.

At each training level, repeat all data-dependent preprocessing using that training subset only, including transcriptome normalization, module definitions and projections, eigengene scaling, topology weights, pooled root-trait scaling, gcPCA extraction and any target scaling. Independent calibration may supply previously frozen quantities. Apply the Clause Zero sign and physiological-window rules in S1.2 without using validation or test outcomes. Standardized RAD RMSE is expressed in units of the relevant training RAD sample standard deviation. For the final independent test, freeze a single development-trained RAD transformation and both fitted predictors. Inner or outer development scores with different fitted target scales are descriptive and do not replace the final paired test estimate.

## S7.3 Paired error difference and practical decision boundary

Here, $\text{ℳ}_{\text{D}\text{=2}}$ and $\text{ℳ}_{\text{D}\text{=3}}$ denote conceptual depth alternatives, not fitted model outputs. For the prospective Equation 6 comparison in evaluation f, calculate both RMSE values on exactly the same held-out observations with the same target, weights and frozen preprocessing. The symbol f identifies an evaluation set, not an independent replicate by virtue of being a fold. In the confirmatory route below, f is the single independent test set. Define the paired difference as follows; positive values favor the higher-capacity comparator. Δ denotes its population counterpart for the stated estimand.

$$\text{Δ}\text{RMSE}_{\text{f}}\text{=}\text{RMSE}_{\text{pairwise,}\text{f}}\text{−}\text{RMSE}_{\text{higher,}\text{f}}\text{ }\text{ }\text{ (}\text{Equation S}\text{23}\text{)}$$

Set δ*>0 before outcome evaluation as the largest acceptable RMSE deficit of the pairwise predictor for a stated biological decision or predictive use. Express it in the same RAD units as the primary RMSE, with the use case, rationale and conversion rule recorded before fitting. Technical-replicate error informs attainable precision, distinguishability and design feasibility; it neither defines nor automatically enlarges δ*. Absence of technical replicates limits the measurement-error audit but is not an automatic prohibition on a utility-based margin or a properly supported predictive comparison. No numerical margin is chosen from the constructed Figure 9 errors; the assumed margin for the conditional design scenarios is defined separately in S7.5.

$$\text{LCB}_{\text{95\%}}\text{(ΔRMSE)>δ*; }\text{UCB}_{\text{95\%, one-sided}}\text{(ΔRMSE)<δ* (Equation S24)}$$

Both bounds in Equation S24 are one-sided 95% bounds (α=0.05). For non-inferiority, H0 is Δ≥δ* and H1 is Δ<δ*; reject H0 only if the upper bound is below δ*. For a practically meaningful higher-capacity advantage, H0 is Δ≤δ* and H1 is Δ>δ*; reject H0 only if the lower bound exceeds δ*. These are directional tests around one practical boundary, not two-sided equivalence. A bound touching δ*, an interval spanning δ*, or unavailable or unreliable uncertainty yields an indeterminate result. A non-significant contrast against zero does not establish adequacy. The protocol must identify the primary comparison and any multiplicity adjustment before examining outcomes.

## S7.4 Uncertainty and the estimand

The primary confirmatory estimand is the difference between the population RMSEs of the two frozen development-trained predictors for new independent units from the declared test population, conditional on the complete development data, fitted RAD transformation, module system and selected models. For clustered sampling, first average squared prediction error within each independent test cluster for each model, then average those cluster losses with equal cluster weights and take the square root separately for each model. Subtract the two RMSEs only after these operations. Thus the estimand gives equal weight to independent clusters; the protocol must justify that weighting for its intended use. A single observation per independent unit is the special case of cluster size one.

Use a paired nonparametric bootstrap of the independent test clusters for the conditional interval. Each bootstrap sample draws the same number of whole clusters with replacement, retains all within-cluster records and both models’ predictions together, and recomputes both RMSEs and their difference. The empirical 5th and 95th percentiles of the bootstrap differences provide the respective nominal one-sided 95% lower and upper bounds. Pre-specify the number of resamples, seed, quantile convention and Monte Carlo precision check. The paired construction retains correlation between model losses and dependence within clusters; it assumes independent, representative clusters and an adequate cluster count. Finite loss variance, non-degenerate RMSEs, cluster imbalance and influential errors must be assessed. Percentile coverage is approximate and is not guaranteed for small or irregular samples; the experiment-specific design must justify and assess it before confirmatory interpretation (Efron and Tibshirani, 1986; Field and Welsh, 2007). S7.5 executes this bootstrap on simulated errors; no empirical transcriptomic comparison is performed.

Repeated or overlapping outer cross-validation folds share training observations and often test observations. They are not independent n and must not supply an ordinary t interval or a standard error based on the fold count (Bengio and Grandvalet, 2004). Retain paired outer predictions as descriptive development diagnostics, keeping each repeat identifiable; do not pool repeated predictions as new independent units. The independent-test bootstrap above does not resample development fits and therefore excludes training, module-selection, target-estimation and tuning uncertainty. Refitting either model or the RAD transformation on the combined development and test data would invalidate the stated conditional interpretation (Bates et al., 2024).

An estimand averaging performance over new development datasets would additionally require repeated complete development and evaluation, including module construction, RAD extraction and inner selection. It needs an experiment-specific full-procedure uncertainty method with its own assumptions and coverage assessment, or independent replication of the complete study. The present protocol does not supply or validate such an interval. Neither the constructed Figure 9 errors nor the frozen-model test interval may be presented as full training-and-selection uncertainty. If only repeated cross-validation is available, this confirmatory Clause B decision remains unevaluated.

## S7.5 Conditional power and test-set size analysis

We evaluated independent test-set planning under the conditional estimand in S7.4. Both predictors, their module universe and the RAD transformation are treated as frozen. Only new test units or clusters vary; no development sample, transcriptomic model or full training pipeline is simulated. Biological application still requires a utility-based margin, an appropriate error model, target power and a complete sampling design fixed before evaluation. The present dimensionless scenarios illustrate numerical planning rather than supply these experiment-specific inputs.

The statistical specification and configuration were frozen and hashed before numerical execution. The higher-capacity predictor has population RMSE 1 on a dimensionless reference scale; the pairwise predictor has population RMSE 1 plus the specified true difference. The illustrative margin is 0.1, with true differences 0, 0.1 and 0.2 for the non-inferiority alternative, shared null boundary and meaningful-advantage alternative, respectively. These round values define the design scenarios, not an estimated or recommended biological tolerance. Figure 9 and the existing geometric analyses supplied no pilot information.

For each independent cluster i and observation j, prediction errors for models k=P,H were generated by the following Gaussian construction. The bivariate cluster effects C_i and observation effects E_ij each have standard-normal marginals and between-model correlation ρ; cluster effects are independent of observation effects, observations are conditionally independent within cluster, and different clusters are independent. Here η denotes within-model correlation between observations in the same cluster, and σ_k is the population error standard deviation.

$$e_{kij}=\sigma_{k}(\sqrt{\eta}C_{ki}+\sqrt{1-\eta}E_{kij}), k\in\{P,H\}$$

The three structures were S1, single observations with ρ=0.7; S2, single observations with ρ=0.3; and S3, four observations per cluster with ρ=0.7 and η=0.5. Errors have mean zero, so population RMSE equals σ_k and the true RMSE difference is known exactly. Gaussian errors allow an analytic check of the generated risks and paired squared-loss moments. The two unit scenarios isolate pairing strength; S3 tests the stated whole-cluster aggregation under repeated observations. It contains 4N observations but only N independent units.

For each structure and N in {20,40,80,160,320,640}, we generated 2,500 independent test sets. Within each set, squared errors were averaged within cluster, then across equally weighted clusters, before taking the square root separately for each model and subtracting. Each interval used 1,999 paired whole-cluster resamples and type-7 quantiles at 0.05 and 0.95. The strict one-sided decision rules in S7.3 were executed in every repetition. No t test or normal-approximation power replaced that procedure.

The three true-difference cases shared standardized errors and bootstrap indices within each structure/N/repetition, giving valid marginal decision rates with common random numbers. Distinct structure/N blocks used separate deterministic seeds. R 4.3.3 used Mersenne-Twister, Inversion normal generation and Rejection sampling; the master seed was 20260906, with data seed equal to the master plus 100000 times the structure index plus 1000 times the N-grid index, and bootstrap seed a further 50000. Both indices start at one. The accelerated index kernel matched R sample.int exactly; fixed-loss RMSE and type-7 quantiles matched direct R calculations. A complete deterministic rerun reproduced all numerical CSVs.

Decision proportions estimate conditional power at the two alternatives and separate directional false-rejection rates at the shared boundary. Their Monte Carlo standard errors use the binomial variance of 2,500 repetitions, and pointwise 95% Wilson intervals quantify simulation uncertainty (Morris et al., 2019). The maximum Monte Carlo standard error is 1 percentage point, and approximately 0.44 percentage points near a 5% rejection rate. Finite-bootstrap randomness is included in the repeated-design operating characteristics; 1,999 resamples provide about 100 draws in each 5% tail. Invalid intervals would count as neither decision and remain in the denominator; none occurred.

Figure S4 plots all frozen structure/N combinations. Table S8 reports N80 and N90 as the first tested N whose raw estimated directional power reaches 80% or 90%; values are not interpolated, smoothed or monotonically adjusted. An unattained target is reported as not reached. Complete grid estimates, Monte Carlo intervals and the first N whose Monte Carlo lower bound reaches each target are also exported; the raw and lower-bound cutoffs coincided wherever reached. Grid resolution and Monte Carlo uncertainty limit any cutoff.

On the tested grid, non-inferiority reached 80%/90% estimated power at N=320/640 for S1 and N=160/320 independent clusters for S3. S2 reached 80% at N=640, but did not reach 90% by N=640. For meaningful advantage, S1 reached both targets at N=640 and S3 at N=320; S2 reached neither by N=640. S3 uses four observations per independent cluster, so its smaller cluster count does not imply fewer total observations. These are nominal power cutoffs for the evaluated procedure, not validated error-controlled sample-size recommendations.

At the shared null boundary and N=20, directional rejection rates ranged from 5.80% to 7.92%; five of the six pointwise 95% Monte Carlo intervals excluded 5% on the high side. At N=640, rates ranged from 5.16% to 5.80%, with all six Monte Carlo intervals including 5%. The small-sample anti-conservatism is retained without changing the interval procedure. Near-nominal results at the largest tested N do not establish coverage for other distributions, cluster structures or sample sizes.

This design analysis evaluates the specified percentile-bootstrap procedure conditionally on two frozen models. It does not estimate training-set or total experimental sample size, validate fitted biological performance, recover interaction order, or assess uncertainty from RAD extraction, module construction or model selection. Biased or heavy-tailed errors, heterogeneous or informative cluster sizes and population shift were not examined. The two boundary rejection rates each target a one-sided 5% level; their union is not claimed to have a 5% family error rate. A real experiment needs its own utility margin and justified error/sampling assumptions, including an assessment of interval reliability before confirmatory use.

## S7.6 Attribution and methodological sources

After an empirical comparison, a declared attribution method may identify hypotheses for biological follow-up. It does not alter U_B or the Clause B decision and cannot identify causal module coordination. Figure 9D retains directly assigned illustrative values rather than a fitted attribution analysis.

The glmnet penalty-factor convention is documented in the official introduction, section Other function arguments, https://glmnet.stanford.edu/articles/glmnet.html (accessed 6 September 2026). Zero factors are unpenalized, and supplied factors are internally rescaled. The original algorithm reference is Friedman et al. (2010), listed in Supplementary References.

## **S8. Sensitivity of RAD-dependent decisions to trait-measurement variation**

Because RAD is derived from image-based root geometric traits, the framework asks whether downstream decisions are stable to plausible measurement variation in the physical inputs. Let $\text{X}^{\left( \text{r} \right)}$ denote the $\text{r}$-th perturbed phenotype matrix. Multiplicative measurement variation is applied as follows.

$$\text{X}^{\left( \text{r} \right)}\text{=}\text{X}\text{∘}\left( \text{1}\text{+}\text{E}^{\left( \text{r} \right)} \right)\text{ }\text{ }\text{ (}\text{Equation S}\text{25}\text{)}$$

Here, $\text{∘}$ denotes element-wise multiplication and $\text{E}^{\left( \text{r} \right)}$ contains zero-centered perturbations whose joint distribution is estimated from repeat scans, segmentations, manual corrections, operators, or batches. Perturbations are applied to the raw four-trait values while preserving treatment labels, experimental-unit matching, and replicate structure. For each perturbed version, pooled standardization and gcPCA extraction are re-fitted and the complete Clause A and Clause B chain is re-run.

Let $\text{I}_{\text{r}}$ indicate whether the focal decision is retained in perturbation run $\text{r}$. The consensus rate is defined as follows.

$$\text{CR=}\frac{\text{1}}{\text{R}}\sum_{\text{r}\text{=1}}^{\text{R}} \text{I}_{\text{r}}\text{ }\text{ }\text{ (}\text{Equation S}\text{26}\text{)}$$

If $\text{CR<0.70}$, the corresponding candidate module-pair term or boundary decision is labeled Measurement-Fragile. A value CR≥0.70 indicates only that the warning was not triggered; it does not establish robustness or biological validation. The continuous consensus value, its uncertainty interval, and a sensitivity curve across empirically estimated error magnitudes are reported. The 0.70 cutoff is an operational warning convention, not a calibrated error-control cutoff or a universal biological threshold.

## **S9. Interpretation boundaries for computational outputs**

The presentation proceeds from RAD extraction and Clause Zero to module continuity, the pairwise basis, Clause A, Clause B and biological follow-up. Fixed-regularization resampling and target-decoy screening nominate terms for Clause A. Clause B independently evaluates the specified pairwise fit over the full outcome-blind U_B against its declared comparator. No candidate-survival gate connects the two questions. A practical-adequacy claim requires the one-sided non-inferiority rule and valid uncertainty for the stated estimand; lack of a detected gain is insufficient. Measurement sensitivity evaluates the effect of root-trait error on these separately defined decisions.

These computational outputs alone identify neither a causal drought-response pathway nor a general interaction-order boundary. They specify candidate-screening and predictive-adequacy questions for future anatomical, physiological, spatial-transcriptomic and perturbational studies.

# 2 Supplementary Figures

Supplementary Figure S1. Descriptive score distributions and restricted-reference alignment (A) The archived full-data cPCA score distributions at γ = 0.5 and 0.7 are retained, including 360 records per treatment and descriptive absolute standardized mean differences of 0.79 and 0.73. This panel is not a new full-data fit. Observation counts are not independent genotype counts. (B) Absolute cosine alignments of source-precision A. tauschii gcPCA and γ = 1 cPCA directions with the frozen global PC1 and PC2 references. The selected-cohort analysis includes all 80 records from 20 accessions. Each local coefficient vector v is mapped as diag(s_global/s_local)v, then restricted to the shared source fields RSA, RV and RD and normalized to unit Euclidean length; the non-equivalent NRL and TRLcalc coordinates are excluded. Global references retain the archived 720-row PCA; they are not re-estimated. This mapping establishes a common coefficient metric for the descriptive comparison. The values are separate and non-additive, not variance capture shares. The historical direct local/global coefficient comparison is replaced; numerical changes in B combine source-precision adoption with this coordinate correction.

# 3 Supplementary Tables

Supplementary Table S1. Descriptive absolute cosine alignments with restricted global PCA references. The two A. tauschii rows use source-precision local coefficients mapped into frozen global pooled-SD coordinates before restriction to RSA/RV/RD and renormalization. **The five full-data cPCA rows retain their archived values and are displayed in increasing γ order.** The restricted references are non-orthogonal; their cosines are non-additive and are not variance-capture shares.

| **Direction** | **Restricted global PC1** | **Restricted global PC2** |
| --- | --- | --- |
| cPCA γ = 0.05 | 0.952169 | 0.556005 |
| cPCA γ = 0.10 | 0.939098 | 0.522144 |
| cPCA γ = 0.20 | 0.808288 | 0.267074 |
| cPCA γ = 0.50 | 0.549713 | 0.853579 |
| cPCA γ = 0.70 | 0.052634 | 0.112575 |
| A. tauschii cPCA gamma=1 | 0.635219 | 0.795854 |
| A. tauschii gcPCA | 0.157117 | 0.191645 |

Supplementary Table S2. Archived full-data cPCA loading vectors across the gamma sweep. Values retain the original additional one-decimal preprocessing and pooled common standardization. For display, eigenvector signs are oriented continuously from γ = 0.70. **TRLcalc replaces the legacy TRL display alias for the operational SRL × RDW product, with unresolved physical units; RD replaces the legacy ARD alias. RSA retains its source identity. Columns follow increasing γ.** These labels do not establish directly measured total root length or mean diameter. The table describes parameter sensitivity, not causal architecture or variance partitioning.

| **Trait** | **0.05** | **0.10** | **0.20** | **0.50** | **0.70** |
| --- | --- | --- | --- | --- | --- |
| TRLcalc | -0.472504 | -0.451367 | 0.358527 | 0.477866 | 0.003181 |
| RSA | -0.592027 | -0.588677 | 0.536273 | -0.066746 | 0.645766 |
| RV | -0.631680 | -0.639675 | 0.615570 | -0.516898 | -0.714699 |
| RD | -0.165000 | -0.201350 | 0.452706 | 0.707111 | 0.268666 |

Supplementary Table S3. Native source-precision A. tauschii loadings at cPCA γ = 1 and for the displayed gcPCA direction.

| **Trait** | **cPCA γ = 1** | Displayed gcPCA | Delta |
| --- | --- | --- | --- |
| TRLcalc | −0.625596 | −0.184879 | 0.440717 |
| RSA | 0.042260 | −0.563656 | −0.605916 |
| RV | 0.644199 | 0.773167 | 0.128968 |
| RD | −0.438009 | −0.224331 | 0.213678 |

Absolute loading cosine = 0.688172; 20 accessions and 80 records. All fields use source precision; TRLcalc = SRL × RDW remains operational and RD retains its source identity. Both vectors have unit length in the native pooled-standardized coordinates. gcPCA is sign-aligned to the RSA-positive cPCA reference, and Delta = gcPCA − cPCA. This display convention does not redefine the frozen RSA-positive response diagnostics.

Supplementary Table S4. Pre-specified constants, scopes, and values intentionally left unset in the prospective framework.

| **Rule or constant** | **Current value or rule** | **Purpose and present scope** | **Before empirical use** |
| --- | --- | --- | --- |
| Pooled preprocessing | One pooled training center and sample-SD transform for both treatments | Source precision for selected-cohort and durum analyses; source-precision accession means for 12-species QC; archived preprocessing for the 720-record descriptive branch | Lock the trait list and transformation before outcome modelling |
| gcPCA ridge | r = ϵ = 1e-6 | Fixed absolute ridge, with complete sensitivity grids retained; no claim that the value makes the fit stable | Report conditioning checks and retain or justify before analysis |
| gcPCA eigenvalue interpretation | λmax≤1 indicates no foreground enrichment under the regularized quotient | Descriptive geometric diagnostic; not a significance test or evidence that a direction is undefined | Report the eigenvalue with the fixed extraction specification. No automatic stopping rule; physiological interpretation requires Clause Zero calibration (S1.3) |
| cPCA diagnostic sweep | Complete frozen registries for Figure 3; γ = 1 loading displays; separate durum γ = 0.7 stability comparator | Geometry within specified settings, not an optimized method-performance benchmark | Do not use it to select RAD; retain fixed gcPCA extraction |
| Continuity weighting | Λ_ij = 1/(1 + ΔR_i + ΔR_j); no fitted rate constant | Candidate-screening penalty, not module identity or preservation | Pre-specify gene set, matching, TOM settings, and retraining scope |
| LASSO lock | 10-fold cross-validation; lambda_min = 0.0542 and lambda_1se = 0.1509 in the controlled scenario | Controlled-scenario lock; prospective Clause A reselects λ for each response | Use the same training-only tuning rule for the observed response and every pseudo-response; lock only within each response's stability subsamples (S6) |
| Stability resampling | S = 100; 50% without replacement; operational retention reference >= 0.60 | Controlled-scenario operational stability; 0.60 is not a calibrated error-control cutoff | Pre-specify sampling unit, seed, iterations, and uncertainty plan |
| Target-decoy display | τ = 0.910 (Figure 7); τ = 0.550 (Figure 8); 25% baseline reference | Operational display and triage settings; q=0.05 in S5 is a nominal count-ratio setting, not an FDR level | Pre-specify the triage rule and empty-set behavior; repeat outcome-derived boundary selection inside every Clause A permutation (S6) |
| Clause A structured null | B = 1,000; discrete upper-tail 95% ceiling (S6) | Prospective full-procedure global-null comparison; not an executed error-control validation | Define the outcome-blind pair family and valid exchangeability blocks; repeat all outcome-dependent operations. Use the discrete ceiling and tie-inclusive tail in S6 |
| Clause B predictive adequacy | Equation 6 fit versus a pre-specified higher-capacity comparator; same outcome-blind U_B and independent test units; Δ = RMSE_pairwise − RMSE_higher on the declared standardized RAD scale | Figure 9 errors and attributions are directly constructed; no fitted comparison or adequacy result. δ* comes from biological or predictive utility, independently of technical-replicate error | Lock all S7 inputs, δ*, model/tuning rules and sampling plan. One-sided α=0.05: LCB>δ* supports meaningful higher-capacity gain; UCB<δ* supports non-inferiority. Conditional paired test-cluster bootstrap; scenario-based numerical planning in S7.5, Figure S4 and Table S8; experiment-specific inputs still required |
| Measurement sensitivity | Measurement-Fragile if consensus <70%; consensus ≥70% means only that the warning was not triggered; empirical error model and repeat count remain protocol-specific | Operational warning convention; no calibrated error-control or empirical robustness status | Estimate the joint four-trait error distribution from repeated scans, segmentations, corrections, operators, or batches before testing |
| Controlled computational topology | Reproducibility seed = 2026; n = 150 samples; p = 770 total features across 10 pre-specified modules (sizes 150, 120, 100, 90, 80, 50, 40, 30, 60, and 50); β = 6; post-TOM display exponent = 0.20 | Controlled computational illustration; no empirical or statistical calibration | Do not reuse as biological or performance evidence |
| Length: raw → analysis → display | Prospective/direct durum: TRL → measured length → TRL (L after standardization). Public workbook: SRL and RDW → SRL × RDW → TRLcalc. | Durum TRL: cm. Public TRLcalc units/calibration remain unresolved; legacy TRL is a display alias. Global NRL is a separate source variable. | Register a directly measured length construct; never equate NRL with TRLcalc by name. |
| Surface: raw → analysis → display | Prospective/direct durum: RSA → directly measured surface area → RSurfA (S after standardization). Public workbook: RSA → source-native RSA → RSA (source field). | Durum RSurfA: cm². Public RSA/RA/TSA mapping is not fully closed; RA remains a distinct source field. Root system architecture is written out. | Confirm the physical construct and unit from primary provenance before treating a public field as surface area. |
| Volume: raw → analysis → display | Prospective/direct durum: RV → measured root volume → RV (V after standardization). Public workbook: RV → source-native RV → RV. | Durum RV: cm³. Public displays retain source identity and do not import durum units. | Register the measurement method and source unit. |
| Diameter: raw → analysis → display | Prospective/direct durum: RD → directly measured mean diameter → RDmean (D after standardization). Public workbook: RD → source root diameter → RD. | Durum RDmean: mm. ARD is the legacy public display alias; it does not establish an average-diameter construct. | Confirm whether diameter denotes a mean, class-specific or other operational measurement. |

Note. Experiment-specific δ* and development-model settings remain unset. S7.5 supplies a conditional numerical design analysis on a dimensionless scale; biological application still requires a justified utility margin, sampling design and frozen models. Technical replicates inform measurement precision and feasibility separately.

## Supplementary Figure S2. Species-level cPCA quality-control screen

(A) Feasibility of the leading cPCA contrast across the frozen 39-value gamma grid for all 12 source-defined species. Blue cells indicate a positive leading contrast eigenvalue and grey cells indicate a non-positive value. Sample sizes are independent accessions; both treatments and both recorded replicates were retained. The red outline and diamond mark the QC-selected *Aegilops tauschii* cohort. (B) Absolute loading-cosine distributions for the selected cohort across 2,000 accession-cluster bootstrap resamples at gamma = 0.70 and 1.00. Violins show the distributions among positive fits; boxes show the interquartile range with 1.5-times-interquartile-range whiskers, and red points mark medians. Labels give the percentage of all bootstrap resamples with a positive leading contrast. (C) Five QC metrics used to compare the complete species cohorts. Red denotes the selected cohort, blue denotes other eligible cohorts, and grey denotes non-eligible cohorts. NA indicates that the gamma = 1 direction was non-positive and a loading cosine was therefore not defined. Eligibility required at least 15 accessions and positive gamma = 1 leading contrasts in the accession-mean and both replication-specific fits. Source data are provided in the accompanying supplementary data package.

Supplementary Figure S3. Durum wheat loading and treatment-response uncertainty. (A) RSurfA-positive gcPCA loading estimates with pointwise 95% complete-refit bootstrap intervals for directly measured TRL, RSurfA, RV and RDmean; RSurfA and RDmean retain the underlying source fields RSA and RD. (B) Equal-genotype mean drought-minus-control contrast in the common coefficient metric. Filled and open wine circles repeat the original estimate, with fixed-axis and complete-refit 95% intervals, respectively. Grey diamonds are all six held-out genotype contrasts, vertically staggered only for visibility; their identities are listed in Table S6c. The experiment includes 36 independent pots, six genotypes, two treatments and three pots per genotype-by-treatment cell. Both interval procedures use the same 2,000 resamples within these cells and remain conditional on the six genotypes. Complete refitting repeats scaling, covariance estimation and axis estimation. The common metric maps training coefficients to the full-panel pooled-SD reference and normalizes their length. It is a post hoc display metric; the primary held-out responses use training-control SD units. The mean contrast is 0.239, with fixed-axis interval [0.183, 0.297] and complete-refit interval [−0.155, 0.312]. The latter includes zero. No interval is estimated from the overlapping held-out folds. All plotted estimates and uncertainty endpoints are unchanged from the frozen analysis. Source: Urbanavičiūtė et al. (2022a), doi:10.3390/agronomy12061329; dataset: Urbanavičiūtė et al. (2022b), doi:10.5281/zenodo.5883299.

## Supplementary Table S5. Complete 12-species cPCA quality-control screen

| Species | Accessions, n | Valid gamma settings | Leading eigenvalue at gamma = 1 | Cosine, gamma 0.70 vs 1.00 | Bootstrap positive, % | Bootstrap cosine, median [2.5th pct] | Replication cosine | QC decision |
| --- | --- | --- | --- | --- | --- | --- | --- | --- |
| Aegilops tauschii | 20 | 39/39 | 0.01350 | 0.990 | 97.65 | 0.991 [0.441] | 0.650 | Selected (rank 1) |
| Triticum durum | 18 | 39/39 | 0.02968 | 0.980 | 99.90 | 0.985 [0.187] | 0.462 | Eligible (rank 2) |
| Aegilops umbellulata | 17 | 39/39 | 0.02008 | 0.728 | 99.10 | 0.905 [0.204] | 0.638 | Eligible (rank 3) |
| Triticum urartu | 17 | 39/39 | 0.00007 | 0.280 | 96.65 | 0.962 [0.017] | 0.862 | Eligible (rank 4) |
| Aegilops triuncialis | 15 | 39/39 | 0.00773 | 0.181 | 99.55 | 0.414 [0.027] | 0.912 | Eligible (rank 5) |
| Aegilops caudata | 7 | 39/39 | 0.62433 | 1.000 | 100.00 | 0.992 [0.249] | 0.076 | Not eligible (rank 6) |
| Aegilops speltoides | 6 | 39/39 | 0.08512 | 0.976 | 100.00 | 0.897 [0.076] | 0.116 | Not eligible (rank 7) |
| Aegilops neglecta | 11 | 36/39 | -0.00001 | NA | 87.10 | NA | NA | Not eligible (rank 8) |
| Triticum boeoticum | 17 | 33/39 | -0.00398 | NA | 79.15 | NA | NA | Not eligible (rank 9) |
| Aegilops cylindrica | 19 | 32/39 | -0.00009 | NA | 70.95 | NA | NA | Not eligible (rank 10) |
| Triticum aestivum | 19 | 30/39 | -0.00004 | NA | 53.55 | NA | 0.133 | Not eligible (rank 11) |
| Aegilops crassa | 14 | 18/39 | -0.00020 | NA | 45.65 | NA | 0.076 | Not eligible (rank 12) |

All 12 complete source-defined species are retained. Bootstrap summaries use 2,000 accession-cluster resamples at gamma = 1; bracketed values give the 2.5th percentile of the absolute loading cosine. NA denotes an undefined cosine for a non-positive nominal contrast. Eligibility and ranking are specified in S1.5.

## Table S6. External durum wheat reference diagnostics

S6a. Raw trait effects. Equal-genotype drought-minus-control mean; pointwise 95% intervals from the frozen 2,000 pot resamples.

| **Trait** | **Unit** | **Mean contrast** | **95% interval** |
| --- | --- | --- | --- |
| TRL | cm | −168.144 | [−248.801, −84.808] |
| RSurfA | cm² | −159.818 | [−181.524, −138.620] |
| RV | cm³ | −5.440 | [−5.949, −4.926] |
| RDmean | mm | −0.441 | [−0.487, −0.391] |

S6b. Geometry and uncertainty. Coefficient conventions and score scales are defined above. A generalized eigenvalue is not a treatment effect.

| **Diagnostic** | **gcPCA** | **cPCA, γ = 0.7** |
| --- | --- | --- |
| Leading eigenvalue | 39.938692 | 0.411147 |
| Second eigenvalue | 0.549694 | −0.024727 |
| Third eigenvalue | 0.014242 | −0.050006 |
| Fourth eigenvalue | 0.005014 | −0.271944 |
| Relative leading gap | 0.986 | 1.060 |
| Background condition number | 1096.345 | 1096.345 |
| Regularized background condition number | 1095.241 | 1096.345 |
| Mean contrast, common metric | 0.239 | −1.370 |
| Mean contrast, training-control SD | 7.467 | −1.672 |
| Projected control SD, native metric | 0.032013 | 0.819300 |
| Fixed-axis 95% interval, common metric | [0.183, 0.297] | [−1.803, −0.931] |
| Complete-refit 95% interval, common metric | [−0.155, 0.312] | [−2.561, 1.222] |
| Fixed-axis 95% interval, control SD | [5.724, 9.277] | [−2.201, −1.136] |
| Complete-refit 95% interval, control SD | [−13.745, 15.353] | [−3.946, 2.707] |
| Bootstrap angle median (degrees) | 1.013 | 13.046 |
| Bootstrap angle 97.5th percentile (degrees) | 41.331 | 47.648 |
| LOGO maximum angle (degrees) | 1.107 | 35.911 |
| LOPO maximum angle (degrees) | 0.830 | 11.051 |
| Held-out signs agreeing with training and baseline | 6 | 4 |

S6c. Held-out genotype responses for gcPCA. Each fit used 30 training and six held-out pots. Common-metric values are descriptive; the primary held-out units use the training-control SD.

| **Held-out genotype** | **Common metric** | **Training-control SD** | **Angle (°)** | **Sign agrees** |
| --- | --- | --- | --- | --- |
| AZEGHAR 2-1 | 0.541 | 16.474 | 0.417 | TRUE |
| CHAM 1 | 0.254 | 8.273 | 0.835 | TRUE |
| JENNAH KHETIFA | 0.068 | 2.453 | 0.803 | TRUE |
| Mv PELSODUR | 0.094 | 3.314 | 0.813 | TRUE |
| SEBATEL 2 | 0.231 | 7.857 | 0.625 | TRUE |
| VULCI | 0.247 | 7.244 | 1.107 | TRUE |

The CSV includes all 12 rows for both methods. No fold-level confidence interval is assigned.

S6d. Complete gcPCA ridge grid. The baseline plus all nine relative-ridge values are retained.

| **Setting** | **Absolute r** | **η** | **λ₁** | **Condition number after ridge** | **Angle (°)** | **Mean contrast, common metric** |
| --- | --- | --- | --- | --- | --- | --- |
| Absolute baseline | 1e-06 | 2.32463e-06 | 39.939 | 1095.241 | 0.000 | 0.239 |
| Relative ridge | 0 | 0 | 39.978 | 1096.345 | 0.001 | 0.239 |
| Relative ridge | 4.30176e-11 | 1e-10 | 39.978 | 1096.345 | 0.001 | 0.239 |
| Relative ridge | 4.30176e-09 | 1e-08 | 39.977 | 1096.340 | 0.001 | 0.239 |
| Relative ridge | 4.30176e-07 | 1e-06 | 39.961 | 1095.870 | 0.000 | 0.239 |
| Relative ridge | 4.30176e-05 | 0.0001 | 38.369 | 1050.793 | 0.029 | 0.239 |
| Relative ridge | 0.000430 | 0.001 | 28.273 | 764.886 | 0.295 | 0.243 |
| Relative ridge | 0.004302 | 0.01 | 8.528 | 206.155 | 2.843 | 0.282 |
| Relative ridge | 0.0430176 | 0.1 | 2.014 | 25.675 | 20.289 | 0.571 |
| Relative ridge | 0.430176 | 1 | 0.804 | 3.519 | 59.247 | −1.275 |

S6e. Frozen stability criteria. These revision-stage rules are conservative working criteria, not validated universal standards. All must hold for the method-specific geometric classification.

| **Method** | **Criterion** | **Observed (source precision)** | **Frozen rule** | **Pass** |
| --- | --- | --- | --- | --- |
| gcPCA | baseline relative gap | 0.986237 | >=0.10 | TRUE |
| gcPCA | bootstrap solved fraction | 1 | >=0.99 | TRUE |
| gcPCA | bootstrap relative gap q025 | 0.597379 | >=0.05 | TRUE |
| gcPCA | bootstrap angle q975 deg | 41.331023 | <=30 | FALSE |
| gcPCA | bootstrap anchor near zero fraction | 0 | <=0.01 | TRUE |
| gcPCA | refit common response CI | -0.154772; 0.312025 | both endpoints strictly on same side of zero | FALSE |
| gcPCA | LOGO heldout sign agreement | 6 | 6/6 agree with own training and full-sample sign | TRUE |
| gcPCA | LOGO max angle deg | 1.106794 | 6 solved and max<=30 | TRUE |
| gcPCA | LOPO max angle deg | 0.829959 | 36 solved and max<=20 | TRUE |
| gcPCA | ridge entire grid max angle deg | 59.246622 | all solved and max<=30 over eta=0..1 | FALSE |
| gcPCA | ridge entire grid response sign | 9 | all grid points agree with baseline sign | FALSE |
| cPCA g0.7 | baseline relative gap | 1.060141 | >=0.10 | TRUE |
| cPCA g0.7 | bootstrap solved fraction | 1 | >=0.99 | TRUE |
| cPCA g0.7 | bootstrap relative gap q025 | 0.716153 | >=0.05 | TRUE |
| cPCA g0.7 | bootstrap angle q975 deg | 47.648135 | <=30 | FALSE |
| cPCA g0.7 | bootstrap anchor near zero fraction | 0 | <=0.01 | TRUE |
| cPCA g0.7 | refit common response CI | -2.561436; 1.222244 | both endpoints strictly on same side of zero | FALSE |
| cPCA g0.7 | LOGO heldout sign agreement | 4 | 6/6 agree with own training and full-sample sign | FALSE |
| cPCA g0.7 | LOGO max angle deg | 35.911440 | 6 solved and max<=30 | FALSE |
| cPCA g0.7 | LOPO max angle deg | 11.050780 | 36 solved and max<=20 | TRUE |

S6f–i. Accompanying source tables in Supplementary_Data_unified/durum_reference/. S6f preserves the original classification in decision_summary.csv. S6g gives all publication-precision checks in paper_table2_reproduction.csv. S6h contains both complete spectra in baseline_spectrum.csv. S6i retains every gamma-registry entry in cpca_gamma_diagnostics.csv and its loadings in cpca_gamma_loadings.csv. The registry retains a near-duplicate 0.7 entry from the original R floating-point construction; entries are not independent replications.

The three publication-mean discrepancies are CHAM 1 drought RSurfA (source RSA; 122.487833 versus 123), SEBATEL 2 control RV (6.047333 versus 6.1), and SEBATEL 2 control RSurfA (source RSA; 264.490833 versus 265).

The cPCA bootstrap and exclusion results in Table S6 concern the frozen primary γ = 0.7 analysis. Main Figure 3D instead shows the original γ = 1 geometric comparator. No γ = 1 bootstrap or stability assessment is inferred from the γ = 0.7 results. Supplementary Figure S3 contains the original gcPCA loading and response intervals.

## Table S6j. Durum γ = 1 geometric comparator

| **Trait** | **cPCA γ = 1** | **Displayed gcPCA** | **Delta** |
| --- | --- | --- | --- |
| TRL | −0.890478 | −0.413217 | 0.477262 |
| RSurfA | 0.042037 | 0.685192 | 0.643155 |
| RV | 0.212405 | 0.014810 | −0.197595 |
| RDmean | −0.400206 | −0.599620 | −0.199414 |

Absolute cosine = 0.639882. This fixed-vector display does not transfer the γ = 0.7 bootstrap results to γ = 1.

## Supplementary Table S7. Frozen source-precision A. tauschii Q1/Q2 diagnostics

| **Diagnostic** | **Source-precision value** |
| --- | --- |
| Leading generalized eigenvalue | 1.898843 |
| Background condition number | 2500.072267 |
| Mean contrast, frozen common metric | 0.143323 |
| Fixed-axis interval lower | 0.101873 |
| Fixed-axis interval upper | 0.185759 |
| Complete-refit interval lower | −0.172771 |
| Complete-refit interval upper | 0.196331 |
| Across-replication response Pearson r | −0.306708 |
| Across-replication response Spearman r | −0.190977 |
| Accessions with the same response sign in both replications | 13.000000 |
| Bootstrap angle median, degrees | 6.346089 |
| Bootstrap angle 97.5th percentile, degrees | 85.824409 |
| Angle after omitting accession 131, degrees | 74.924859 |
| Replication 1 train to replication 2 test, training-control SD | 3.507517 |
| Replication 2 train to replication 1 test, training-control SD | −1.996196 |

The common reference SDs for the response and angular diagnostics are those frozen in the preceding audit, not the native plotting coordinates or the global PCA reference. The full 68-metric old/source-precision comparison and response-interval source table accompany this table. No new resampling, exclusion or ridge analysis was performed. The two observed replications do not support a claim of general repeatability or heritability.

Table S7 numerical sources are in Supplementary_Data_unified/source_precision_tauschii: baseline_spectrum.csv and baseline_geometry.csv give all four generalized eigenvalues, eigengaps and background conditioning; analysis_inputs_source_precision.csv and cohort_source_precision.csv identify all 80 records; matched_responses.csv and accession_response_means.csv provide all 40 matched contrasts and 20 accession means; matched_response_summary.csv and fixed_panel_variance_decomposition.csv give the conditional model; comparison_response_intervals.csv gives all old/source interval endpoints; bootstrap_draws.csv, bootstrap_loadings.csv and bootstrap_loading_intervals.csv retain the 2,000 complete-refit results; loao_geometry_response.csv and loao_heldout_responses.csv give all 20 exclusion fits; replication_geometry.csv and replication_cross_projection.csv give both replication fits and cross-projections; ridge_sensitivity.csv retains the complete grid including zero and η = 1. TableS7_all_68_frozen_diagnostics.csv in updated_figure_and_registry_tables retains every old/source summary metric. These are frozen numerical outputs, not additional analyses performed for manuscript assembly.

Supplementary Figure S4. Conditional power and sample-size planning for Clause B. (A) Power matrix for non-inferiority at true Δ=0 and meaningful higher-capacity advantage at Δ=0.2, with the illustrative margin δ*=0.1 and higher-model population RMSE 1. Cell shading and bold numbers give power (%); brackets give pointwise 95% Wilson Monte Carlo intervals. (B) The two directional rejection rates are shown separately at the shared null boundary Δ=δ*, with a dashed 5% reference. Circles and triangles denote non-inferiority and higher-capacity advantage, respectively. S1 and S2 use independent single observations with between-model error correlations 0.7 and 0.3, respectively. S3 uses four observations per independent cluster, correlation 0.7 between models and within-model intra-cluster correlation 0.5. All errors follow the zero-mean Gaussian construction in S7.5. Each estimate uses 2,500 independently generated test sets and 1,999 paired whole-cluster percentile-bootstrap samples per set, with type-7 quantiles and the strict one-sided decision rules. Horizontal intervals in B show the same type of Monte Carlo interval, not RMSE prediction intervals. Small vertical offsets separate the two decision rules at the same N in B. N counts independent test units or clusters; S3 contains 4N observations. The figure-level label identifies conditional simulation with frozen predictors. No empirical transcriptomic model was fitted; these scenarios do not estimate fitted transcriptomic performance or recommend a biological margin. The two directional boundary errors do not imply a combined 5% error guarantee.

## Supplementary Table S8. Conditional test-set planning under the frozen error scenarios.

| **Scenario / error structure** | **Decision** | **N80** | **N90** | **Boundary, N=20 % ± MCSE (pp)** | **Boundary, N=640 % ± MCSE (pp)** |
| --- | --- | --- | --- | --- | --- |
| S1: m=1, ρ=0.7 | NI | 320 | 640 | 7.92 ± 0.54 | 5.80 ± 0.47 |
| S1: m=1, ρ=0.7 | Advantage | 640 | 640 | 5.80 ± 0.47 | 5.16 ± 0.44 |
| S2: m=1, ρ=0.3 | NI | 640 | NR | 7.32 ± 0.52 | 5.36 ± 0.45 |
| S2: m=1, ρ=0.3 | Advantage | NR | NR | 7.24 ± 0.52 | 5.16 ± 0.44 |
| S3: m=4, ρ=0.7 η=0.5; 4N observations | NI | 160 | 320 | 7.04 ± 0.51 | 5.64 ± 0.46 |
| S3: m=4, ρ=0.7 η=0.5; 4N observations | Advantage | 320 | 320 | 6.36 ± 0.49 | 5.40 ± 0.45 |

NI denotes non-inferiority at true Δ=0; advantage uses Δ=0.2. The dimensionless margin is 0.1 and the higher-model population RMSE is 1. All errors are unbiased Gaussian. m is the number of observations per independent cluster, ρ the between-model error correlation and η the within-model intra-cluster correlation. N80/N90 are the first tested independent-unit counts reaching raw estimated 80%/90% power; NR means not reached by N=640. No interpolation was used. Boundary entries at Δ=0.1 are rejection percentages ± Monte Carlo standard error in percentage points, based on 2,500 repetitions. The full grid and pointwise 95% Monte Carlo intervals accompany the table in CSV. Elevated small-N errors limit interpretation of these nominal power cutoffs; they are not validated sample-size recommendations. S3 counts clusters, with 4N total observations.

## Supplementary References

Bates, S., Hastie, T., and Tibshirani, R. (2024). Cross-validation: What does it estimate and how well does it do it? Journal of the American Statistical Association 119, 1434–1445. doi:10.1080/01621459.2023.2197686.

Bengio, Y., and Grandvalet, Y. (2004). No unbiased estimator of the variance of K-fold cross-validation. Journal of Machine Learning Research 5, 1089–1105. https://www.jmlr.org/papers/v5/grandvalet04a.html.

de Oliveira, E. F., Garg, P., Hjerling-Leffler, J., Batista-Brito, R., and Sjulson, L. (2025). Identifying patterns differing between high-dimensional datasets with generalized contrastive PCA. PLOS Computational Biology 21, e1012747. doi: https://doi.org/10.1371/journal.pcbi.1012747.

Efron, B., and Tibshirani, R. (1986). Bootstrap methods for standard errors, confidence intervals, and other measures of statistical accuracy. Statistical Science 1, 54–75. doi:10.1214/ss/1177013815.

Field, C. A., and Welsh, A. H. (2007). Bootstrapping clustered data. Journal of the Royal Statistical Society: Series B (Statistical Methodology) 69, 369–390. doi:10.1111/j.1467-9868.2007.00593.x.

Friedman, J., Hastie, T., and Tibshirani, R. (2010). Regularization Paths for Generalized Linear Models via Coordinate Descent. Journal of Statistical Software 33, 1–22. doi: https://doi.org/10.18637/jss.v033.i01.

Langfelder, P., Luo, R., Oldham, M. C., and Horvath, S. (2011). Is My Network Module Preserved and Reproducible? PLoS Computational Biology 7, e1001057. doi: https://doi.org/10.1371/journal.pcbi.1001057.

Morris, T. P., White, I. R., and Crowther, M. J. (2019). Using simulation studies to evaluate statistical methods. Statistics in Medicine 38, 2074–2102. doi:10.1002/sim.8086.

Pour-Aboughadareh, A., and Poczai, P. (2021b). A dataset on multi-trait selection approaches for screening desirable wild relatives of wheat [Data set]. Version 1. Mendeley Data. doi: https://doi.org/10.17632/84kmmmgvvr.1.

Urbanavičiūtė, I., Bonfiglioli, L., and Pagnotta, M. A. (2022a). Diversity in Root Architecture of Durum Wheat at Stem Elongation under Drought Stress. Agronomy 12, 1329. doi: https://doi.org/10.3390/agronomy12061329.

Urbanavičiūtė, I., Bonfiglioli, L., and Pagnotta, M. A. (2022b). Raw data: Diversity in root architecture of durum wheat at stem elongation under drought stress [Data set]. Version 1.0. Zenodo. doi: https://doi.org/10.5281/zenodo.5883299.

Winkler, A. M., Ridgway, G. R., Webster, M. A., Smith, S. M., and Nichols, T. E. (2014). Permutation inference for the general linear model. NeuroImage 92, 381–397. doi: https://doi.org/10.1016/j.neuroimage.2014.01.060.
